# CD99 contributes to the EWS::FLI1 transcriptome by specifically affecting FOXM1‐targets involved in the G2/M cell cycle phase, thus influencing the Ewing sarcoma genetic landscape

**DOI:** 10.1002/ccs3.12047

**Published:** 2024-08-02

**Authors:** Michela Pasello, Maria Antonella Laginestra, Maria Cristina Manara, Lorena Landuzzi, Francesca Ruzzi, Margherita Maioli, Evelin Pellegrini, Alessandra De Feo, Pier‐Luigi Lollini, Katia Scotlandi

**Affiliations:** ^1^ Laboratory of Experimental Oncology IRCCS Istituto Ortopedico Rizzoli Bologna Italy; ^2^ Laboratory of Immunology and Biology of Metastasis Department of Medical and Surgical Sciences (DIMEC) University of Bologna Bologna Italy; ^3^ Department of Pathology IRCCS Istituto Ortopedico Rizzoli Bologna Italy

**Keywords:** biomarkers, CD99, Ewing sarcoma, EWS::FLI1, transcriptome, tumor growth

## Abstract

Ewing sarcoma (EwS), a highly aggressive malignancy affecting children and young adults, is primarily driven by a distinctive oncogenic fusion, the EWSR1‐ETS, whose activity is a key source of epigenetic and clinical heterogeneity. CD99 is constantly present in EwS cells, known to modulate the EwS genetic profile and tumor malignancy. However, the relevance of CD99 alone, or in association with EWSR1‐ETS chimeras, is poorly understood. We explored the dynamic relationship between CD99 and EWS::FLI1, the main fusion observed in EwS, by means of model systems with inducible expression of either molecule. The transcriptomic dynamics of cells with or without expression of EWS::FLI1 or CD99 were analyzed and correlated with tumor cell growth. The CD99‐associated EwS gene profile was found to have commonalities with the profile induced by EWS::FLI1, but also peculiar differences. Both EWS::FLI1 and CD99 are regulated targets of the DREAM complex, but the CD99 expression specifically impacted genes that are the targets of FOXM1 and are involved in the setting of the G2/M phase of the cell cycle. Most CD99‐regulated FOXM1‐targeted genes were found to correlate with bad prognosis in two public clinical datasets (R2 platform), further supporting the clinical relevance of CD99‐mediated regulation of EwS gene expression.

## Graphical abstract

Time course analysis of differential gene expression in Ewing sarcoma experimental models with inducible expression of EWS::FL1 or CD99. The analysis revealed up‐regulation of genes involved in the DREAM complex target genes either when the chimera or the CD99 antigen are inhibited. However, most of CD99‐regulated genes are specifically involved in the setting of the G2/M phase of the cell cycle and are targets of the FOXM1 transcription factor. Eleven of them were found to correlate with poor prognosis in clinical samples.

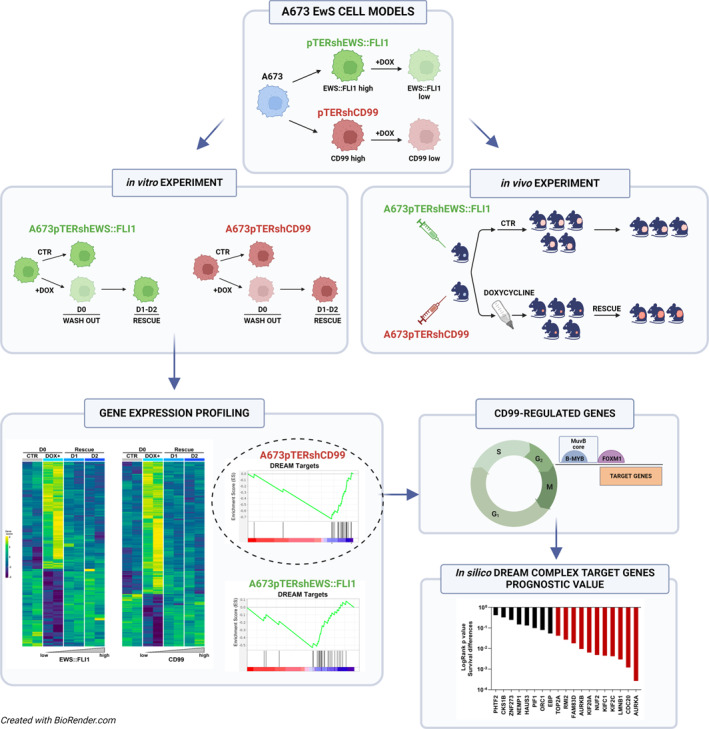

## 1 INTRODUCTION

Ewing sarcoma (EwS) is an aggressive childhood malignancy of bone and soft tissues, with a high metastatic propensity. The standard of care is a multimodal treatment regimen, including surgical resection and/or local radiotherapy as well as intensive multi‐agent chemotherapy, which is effective for patients with localized disease. However, patients with metastases at diagnosis and patients who do not respond to first line therapy have disappointingly low survival.^1^, ^2^, ^3^ To identify novel therapeutic approaches, a deeper understanding of the mechanisms leading to the malignant progression of EwS is needed.

EwS is presumed to originate from an undifferentiated stem cell with features of both mesenchymal and neural lineage, and it is characterized by a high level of epigenetic heterogeneity and plasticity, while its genome is stable, with rare recurrent mutations.^4^, ^5^ EwS is driven by a specific chromosomal translocation that fuses a member of the FET family of RNA‐binding proteins involved in transcription and splicing (FUS, EWSR1, or TAF15), with an ETS family transcription factor. The most common chimera EWS::FLI1 (85% of EwS cases) is known to induce malignant transformation by regulating a variety of molecular processes including chromatin architecture, gene transcription, RNA splicing, R‐loop formation, and protein translation.^6^ Even though it is driven by a single genetic mutation, EwS is endowed with a considerable clinical heterogeneity. The variability of EWS::FLI1 expression and transcriptional activity among individual tumor cells is emerging as a critical determinant of epigenetic heterogeneity, tumor cell phenotype, and disease progression.^7^, ^8^

EwS cells are also characterized by the peculiarly high expression of CD99, a cell surface molecule that is involved in the regulation of crucial biological processes. EwS cells deprived of CD99, but still expressing EWS::FLI1, showed dramatically inhibited growth, migration, and metastatic capabilities and tended to differentiate toward the neural lineage.^9^, ^10^ Furthermore, CD99 depletion from the cell surface induced transcriptional dysregulation through the zyxin–GLI1 axis, specifically affecting the expression of crucial EWS::FLI1 targets.^11^, ^12^

Available data suggest a possible link between EWS::FLI1 and CD99, but no clear evidence has emerged so far.^13^, ^14^ Regulation of CD99 expression by EWS::FLI1, either directly (through binding to the promoter)^9^ or indirectly (through miRNAs)^7^ has been reported, however the silencing of EWS::FLI1 in EwS cells did not result in a significant decrease in CD99 expression. To investigate the relationship between EWS::FLI1 and CD99, we developed experimental models with inducible silencing of either EWS::FLI1 or CD99, and we analyzed the gene expression profiles associated with the silencing and/or the recovery of CD99 and EWS::FLI1 expression, both in vitro and in vivo.

## 2 MATERIALS AND METHODS

### 2.1 EWS::FLI1 and CD99 tetracycline‐inducible systems

A673 (RRID:CVCL_0080) cells were purchased from American Type Culture Collection (ATCC) and transfected with the plasmid pcDNA/6TR (Thermo Fisher Scientific) encoding the reverse tetracycline (TET)‐responsive transcriptional activator^15^ and with the plasmid pTER/shEWS::FLI1 (kindly provided by K. Laud‐Duval, Institut Curie, Paris)^16^ or the plasmid pTER/shCD99 (engineered in our laboratory). The transfected cells were selected with blasticidin (2 μg/mL, #R21001, Thermo Fisher Scientific) and zeocin (50 μg/mL, #R25005, Thermo Fisher Scientific) and named A673pTERshEWS::FLI1 or A673pTERshCD99. To avoid cross‐contamination between cell lines and outgrowth of faster‐growing clones in long‐term cultures, all cell lines were kept in liquid nitrogen until use. When in culture, they were maintained in Iscove's modified Dulbecco's medium (IMDM, #ECB2072L, EuroClone) supplemented with 10% fetal bovine serum (FBS, #ECS0180L, EuroClone) or with 10% Tet System Approved FBS (#631106, Takara) and incubated at 37°C in a humidified atmosphere containing 5% CO_2_ for approximately 8–12 in vitro passages (corresponding to 2–3 months) before being discarded. Whenever necessary, replicates started from the same batch of frozen vials. Cells were regularly tested for *Mycoplasma* contamination (MycoAlert *Mycoplasma* Detection Kit, #LT07–418, Lonza) and authenticated by short tandem repeat PCR analysis (CLA service by Eurofins Genomics; last control July 2023). EWS::FLI1 or CD99 silencing was achieved by adding 2 μg/mL of doxycycline (DOX; #D9891, Sigma Aldrich) in the cell culture medium for 48 h. After 48 h, silenced (DOX+) and CTR cells were collected and labeled as day 0 (D0). To allow the re‐expression of EWS::FLI1 or CD99, in some cultures DOX was removed from the medium, cells were then collected from day 1 to day 14 (D1‐D14).TC‐CD99‐shRNA, BRZ‐CD99‐shRNA and CAR‐CD99‐shRNA silenced models for CD99, previously obtained in our laboratory,^9^, ^17^ were used for validation.

### 2.2 RNA extraction and real‐time quantitative reverse transcription‐PCR

Total RNA was extracted using the Trizol Reagent (#15596018, Thermo Fisher Scientific) and reverse‐transcribed into cDNA using a High‐Capacity cDNA Reverse Transcription Kit (#4368813, ThermoFisher Scientific) according to the manufacturer's protocols. RT‐qPCR was performed on a ViiA7 system (ThermoFisher Scientific). We used TaqMan PCR Master Mix (#4364340, Thermo Fisher Scientific) and predesigned assays for *EWS*:*:FLI1* (Fw: 5′‐GGC CAG AAT TCA TGT TAT TGC‐3’; Rev: 5′‐CCA AGT CAA TAT AGC CAA CAG‐3’; Probe: 5′‐56‐FAM/ACG GGC AGC AGA ACC CTT CTT AT/36‐TAMSp‐3′, IDT), *CD99* (Hs00908458_m1, ThermoFisher Scientific) and (Fw: 5′‐GAA GGT GAA GGT CGG AGT‐3’; Rev: 5′‐GAA GAT GGT GAT GGG ATT TC‐3’; Probe: 5′‐56‐FAM/CAA GCT TCC CGT TCT CAG CC/36‐TAMSp‐3′, IDT). SYBR Green PCR Master Mix (#4309155, Thermo Fisher Scientific) was used for *FOXM1*, *AURKA*, *FAM83D*, *KIF20A*, *KIF2C*, *LMNB1*, *and NUF2* (primers are listed in the Table S1). The expression levels of target genes were normalized to that of GAPDH and expressed as 2^−ΔΔCt.^18^

### 2.3 Western blotting

Western blotting (WB) was performed according to standard protocols. Equivalent amounts of protein collected at the different time points were separated by electrophoresis on a 4%–15% resolving gel (Mini‐PROTEAN™ TGX Stain‐Free™ Protein Gels; #4568083, Bio‐Rad Laboratories Inc.) and transferred to nitrocellulose membranes. Membranes were incubated overnight with primary antibodies: anti‐FLI1 (#ab15289, rabbit polyclonal, Abcam), anti‐CD99 (12E7, #sc‐53148, mouse monoclonal, Santa Cruz Biotechnology), anti‐FOXM1 (EPR17379, #ab207298, rabbit monoclonal, Abcam), and anti‐cyclinB1 (H‐433, #sc‐752, rabbit polyclonal, Santa Cruz Biotechnology). Horseradish peroxidase (HRP)–conjugated donkey anti‐rabbit (#NA9340, GE Healthcare) and sheep anti‐mouse (#NA9310, GE Healthcare) secondary antibodies were used. Proteins were visualized with Clarity Western ECL Substrate (#1705061, Bio‐Rad Laboratories Inc.) and images were analyzed with Image Lab system (ChemiDoc MP, Bio‐Rad Laboratories Inc.).

### 2.4 In vitro tumor cell growth

Cells were plated into 6‐well plates (200.000 cells/well) in medium with 10% Tet System Approved FBS and cultured for 24 h before being treated with 2 μg/mL of DOX. After 48 h (D0), DOX was removed from the medium to obtain progressive re‐expression of EWS::FLI1 or CD99 from D1 to D6. Tumor cell growth was detected at each time point by trypan blue vital counting (#T8154, Sigma‐Aldrich).

### 2.5 In vivo studies

Female NOD Scid gamma (NSG) mice, 13–25 weeks old (breeders obtained from Charles River), received a subcutaneous (s.c.) injection of 0.5 × 10^6^ A673pTERshEWS::FLI1 or A673pTERshCD99 cells. Tumor growth was measured with calipers twice weekly; tumor volumes were calculated as π/2[√(a·b)]3/6], where a and b are the two maximal perpendicular diameters. For in vivo gene silencing, groups of eight mice received either 2 mg/mL DOX in 5% sucrose (#S9378, Sigma Aldrich)^19^ or 5% sucrose only, in autoclaved drinking water, starting from five days after cell injection, for 17 days. Drinking water was protected from light and changed every 3–4 days. Mouse body weights were measured at least once a week; no significant variation in body weight was observed among the groups at the end of the treatment.

Two mice per group were sacrificed, during gene silencing, on day 22 after cell injection. The remaining six mice per group were followed for eight additional days after DOX removal to evaluate the re‐activation of gene expression and the rescue of tumor growth. When tumors exceeded the volume of 2.5 cm^3^, mice were sacrificed for ethical reasons using CO_2_ inhalation and cervical dislocation. An accurate necropsy was performed. Tumor samples for histopathology and immunohistochemistry were fixed in 10% buffered formalin and embedded in paraffin; samples for molecular studies were snap frozen in liquid nitrogen and stored at −80C.

### 2.6 Immunohistochemistry

Serial 3‐μm‐thick tissue sections from formalin‐fixed, paraffin‐embedded xenografts were processed according to standardized immunohistochemical procedures and then immunostained with the following primary antibodies: anti‐FLI1 (C‐19, #sc‐356, rabbit polyclonal, Santa Cruz Biotechnology), anti‐CD99 (O13, #915601, mouse monoclonal, BioLegend), anti‐FOXM1 (EPR17379, #ab207298, rabbit monoclonal, Abcam), and anti‐cyclinB1 (H‐433, #sc‐752, rabbit polyclonal, Santa Cruz Biotechnology). An avidin–biotin–HRP method was used for staining (VECTASTAIN® ABC kit, #PK‐4002, or VECTASTAIN® ABC kit, #PK‐4001, Vector Laboratories). For morphological analyses, one slide for each xenograft was stained with hematoxylin and eosin. Histological and histomorphometric analyses were carried out with Nikon 90i Eclipse with a plan Fluor 40x DIC M, N.A. 0.75, Refractive Index: 1. Images of 2560 × 1920 pixels were collected using a Nikon DS‐U2/L2 USB digital camera and rendered using NIS Elements software (Nikon).

### 2.7 Microarray experiment and data analysis

Gene expression profiles of two biological replicates from A673pTERshEWS::FLI1 and A673pTERshCD99 cells were assessed by microarray profiling using Human Gene Expression Microarrays (SurePrint G3 Human Gene Expression v3 8 × 60K Microarray Kit, #G4851C, Agilent Technologies) according to the manufacturer's procedures. Images at 5 μm resolution were generated by the Agilent scanner and Feature Extraction 10.5 software (Agilent Technologies) was used to obtain raw expression data, which were quantile‐normalized and log_2_‐transformed using single‐channel Agilent microarray pre‐processing analysis by the limma Bioconductor package.^20^ To explore the transcriptional dynamics of CD99 or EWS::FLI1 expression we performed time course differential expression analysis (D0, D1, and D2) implemented in the limma package.^20^ We set up the matrix design to identify which genes responded differently over time as follow: DEG < ‐ makeContrasts (“DOX + vs. CTR,” “D1 vs. D0,” “D2 vs. D1,” levels = design). Genes whose expression changed over time (from up‐regulated to down‐regulated or vice versa) after DOX removal at D1 and D2, were considered significant at *p*‐values ≤0.05 and absolute |log_2_FC |>1. Heatmap of their expression was visualized by ComplexHeatmap R package. Two published microarray‐based data related to gene expression profile of TC‐71 and IOR/BRZ Ewing sarcoma cells stably modified for CD99 expression (GSE10993^9^) were used to test the robustness of the A673pTERshCD99 signature. Raw data were quantile‐normalized and log_2_‐transformed using single‐channel Agilent microarray pre‐processing analysis by limma Bioconductor package.^20^ To investigate the role of the A673pTERshCD99 signature in EwS models, we also considered two in silico microarray gene expression datasets: 1) UET‐13 cells exploiting tetracycline‐inducible system for EWS::FLI1 expression (GSE8665^13^) and 2) EWS::FLI1‐silenced EwS cell lines (GSE7007^16^). Raw CEL file data were normalized using robust multi‐array average normalization (RMA) and log_2_ transformed. Unsupervised hierarchical clustering (HC) using the Pearson correlation as distance measure and Ward.D2 as clustering method was adopted. Z‐scores of log_2_ transformed expression values were displayed by the ComplexHeatmap R package.^21^ Clustering of differentially expressed genes was performed using short‐time series expression miner algorithm (STEM).^22^ Log_2_ gene expression values were used for clustering with default parameter settings, except for the minimum correlation of profile, which was set to 0.9, to group similar expression profiles. The clusters were displayed as transcript‐wise Z score of log_2_ expression values using ggplot2 R package.^23^ Functional analysis of the differentially expressed genes over time was performed by gene set enrichment analysis (GSEA)^24^ using a continuous phenotype label to analyze time series data and the Molecular Signature Database (MSigDB 2023.1 released) c2.all.v7.0.symbols.gmt signature. For time series data, Pearson's correlation was selected in the metric for ranking genes. The most relevant gene sets were selected considering a normalized enrichment score (NES) ≥ 1.8 and a *p*‐value ≤0.01.

### 2.8 Statistical analyses

Statistical analyses were performed using non‐parametric tests (Kruskal–Wallis for unpaired data and Mann–Whitney *U* test for unpaired two‐group data). For in vivo studies, multiple comparisons statistical analyses were performed using two‐way ANOVA with Bonferroni post‐test; unpaired *t* test with Welch's correction was used for comparison between two groups having variances significantly different. The values were expressed as mean ± SD. The differences with *p* < 0.05 were considered statistically significant. GraphPad Prism version 10 was used for statistical analyses. The R2 Genomics Analysis and Visualization platform (Academic Medical Center, Amsterdam, The Netherlands; R2: Genomics Analysis and Visualization Platform; http://r2.amc.nl) was used to generate Kaplan–Meier overall survival (OS) curves. We used “Mixed Ewing Sarcoma—Savola—117—MAS5.0—u133p2” (GSE17618 dataset) and “Tumor Ewing Sarcoma (Core Transcript)—Dirksen—85—rma_sketch—huex10t” (GSE63157^25^). The R2 generated “scan” cut‐off modus was used to determine the threshold point that most significantly separates high relative gene expression versus low relative gene expression. All factors significantly associated with OS in univariate analysis were entered into a Cox proportional hazards model multivariate analysis. Values of 95% confidence intervals (CI) of hazard ratios (HR) were provided.^26^

## 3 RESULTS

### 3.1 Generation of shEWS::FLI1 or shCD99 inducible A673 models

**FIGURE 1:**
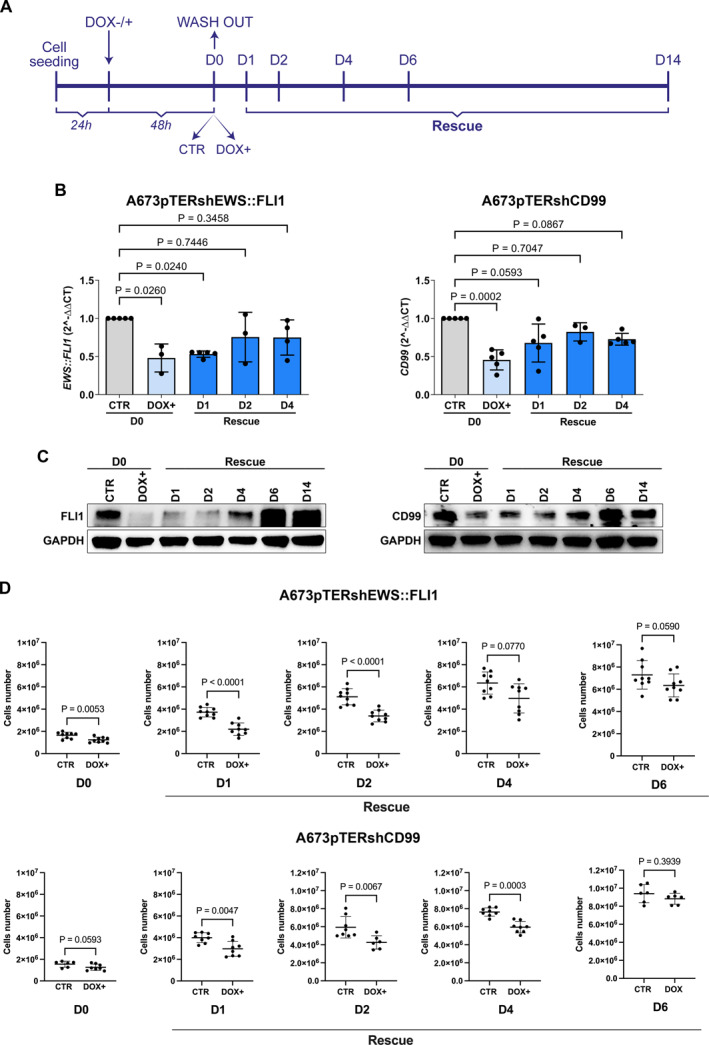
Inducible A673 in vitro models expressing shEWS::FLI1 or shCD99. (A) Schematic representation of the experimental design. (B) Expression level of *EWS*:*:FLI1* and *CD9*9 by RT‐qPCR in A673pTERshEWS::FLI1 and A673pTERshCD99 cells respectively at the indicated time point. Data are expressed as mean ± SD (Kruskal–Wallis test). (C) Protein expression by Western Blot of FLI1 and CD99 in A673pTERshEWS::FLI1 and A673pTERshCD99 respectively; GAPDH was used as a loading control. Representative blots from three independent experiments are shown. (D) Inhibition of in vitro proliferation of A673pTERshEWS::FLI1 and A673pTERshCD99 cells after doxycycline (DOX) induced gene silencing (D0) and after DOX withdrawal from D1 to D6 (rescue). Data are expressed as mean ± SD, statistical comparisons were made by the Mann–Whitney *U* test.

To investigate whether and how EWS::FLI1 and CD99 interacted in the regulation of gene expression, we created variants of the A673 EwS cell line using a tetracycline‐inducible shRNA system targeting EWS::FLI1 or CD99 transcripts (named A673pTERshEWS::FLI1 or A673pTERshCD99, respectively). Silencing of EWS::FLI1 or CD99 was obtained by adding DOX to the culture medium for two days and then collecting the cells (day 0, D0). Rescue time‐dependent experiments were then performed from day 1 to day 14 by withdrawing DOX from the culture medium (D1 to D14). The experimental approach is summarized in Figure 1A. At the transcriptional level, silencing was quickly reversed, after 24 h (D1) for CD99 and after 48 h (D2) for EWS::FLI1 (Figure 1B); at the protein level, expression of EWS::FLI1 and CD99 was completely restored after 6 days (D6, Figure 1C).

**FIGURE 2:**
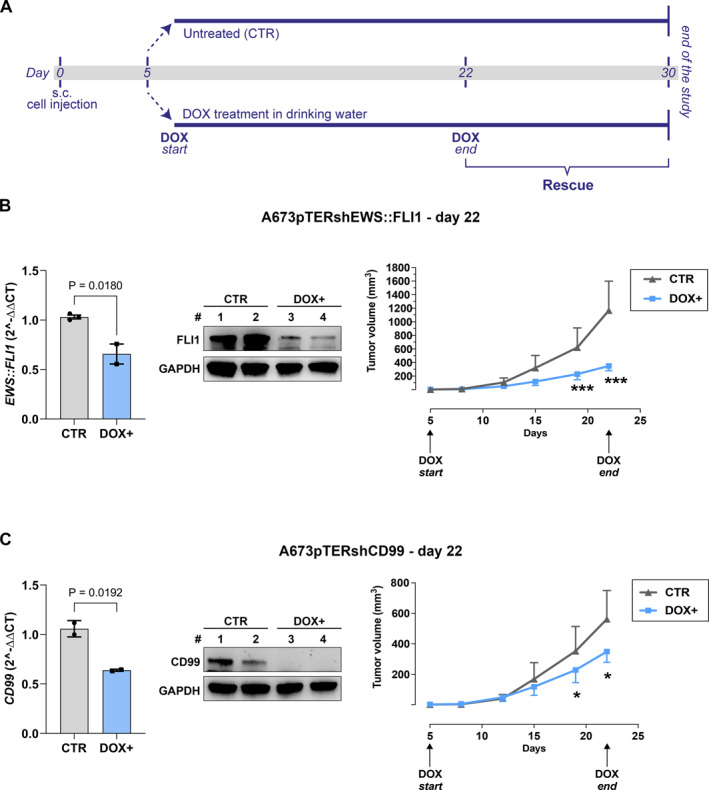
Inhibition of tumor growth in inducible silencing of EWS::FLI1 or CD99. (A) In vivo experimental design. Tumor growth was measured during doxycycline (DOX) treatment from day five and to day 22 after cell injection, when 2‐3 mice per group were used to evaluate gene and protein expression. (B) From left to right: *EWS*:*:FLI1* mRNA expression analysis by RT‐qPCR in A673pTERshEWS::FLI1 xenografts with or without DOX induction, each bar represents the mean ± SD, (unpaired *t* test); FLI1 protein expression by Western Blot in A673pTERshEWS::FLI1 xenografts with or without DOX induction, GAPDH was used as a loading control; growth of A673pTERshEWS::FLI1 tumors, each point represents mean tumor volumes (*n* = 8), bars indicate SD (***p < 0.0001, two‐way ANOVA with Bonferroni post‐test). (C) From left to right: *CD99* mRNA expression analysis by RT‐qPCR in A673pTERshCD99 xenografts with or without DOX induction, each bar represents the mean ± SD, (unpaired *t* test); CD99 protein expression by Western Blot in A673pTERshCD99 xenografts with or without DOX induction. GAPDH was used as a loading control; tumor growth of A673pTERshCD99 cells, each point represents mean tumor volumes (*n* = 8), bars indicate SD (*p < 0.05, two‐way ANOVA with Bonferroni post‐test).

**FIGURE 3:**
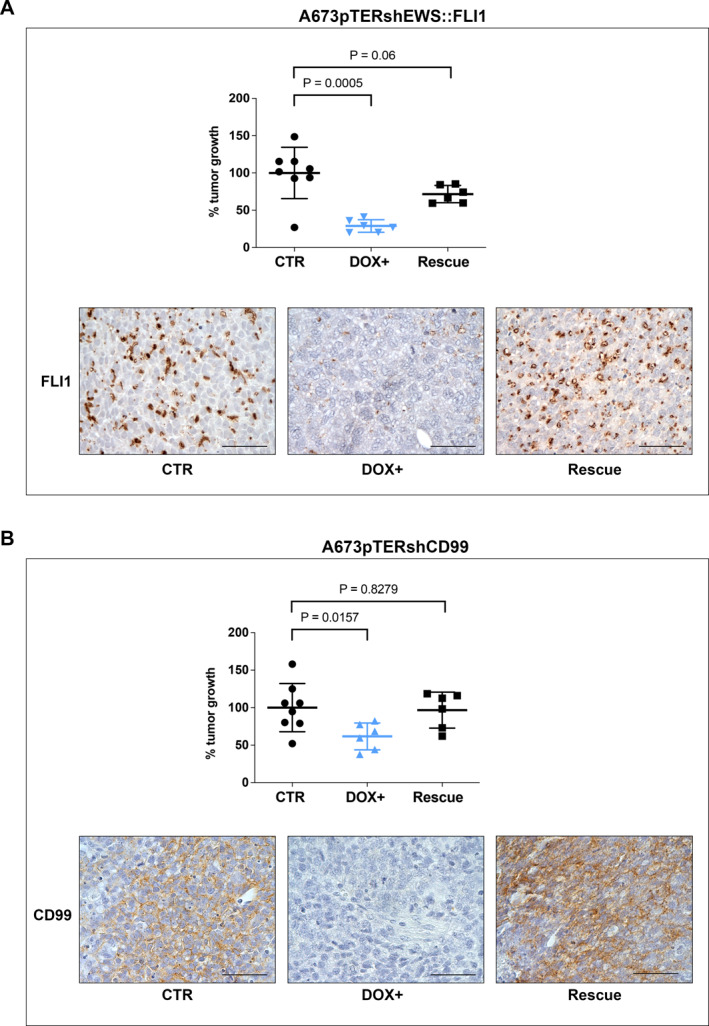
Rescue of tumor growth and protein level after doxycycline withdrawal. (A) A673pTERshEWS::FLI1; (B) A673pTERshCD99. Six mice per group were followed before and after doxycycline (DOX) removal. Each point represents percent tumor growth over the mean tumor volume of untreated control mice at the corresponding time points, values were significantly lower during DOX treatment, but not significantly different from untreated mice after DOX removal (Rescue, unpaired *t* test with Welch's correction), lines represent mean ± SD. Immunohistochemistry staining of FLI1 in A673pTERshEWS::FLI1, and of CD99 in A673pTERshCD99 are shown (scale bar, 50 μm).

Knock‐down (KD) of both EWS::FLI1 and CD99 significantly impaired EwS cell growth in vitro (Figure 1D) and tumor growth in vivo (Figure 2). NSG mice bearing measurable s.c. A673pTERshEWS::FLI1 or A673pTERshCD99 tumors were randomized to receive or not DOX in the drinking water (Figure 2A). As long as DOX was present, the expression of EWS::FLI1 or CD99 was silenced, and tumors grew significantly less than untreated controls (Figure 2B,C). When DOX was withdrawn, the expression of EWS::FLI1 and CD99 was rescued (Figure 3), tumor growth restarted and became similar to that of controls (Figure 3A,B).

### 3.2 CD99‐mediated transcriptome participates with EWS::FLI1‐mediated gene expression to the regulation of EwS malignancy

**FIGURE 4:**
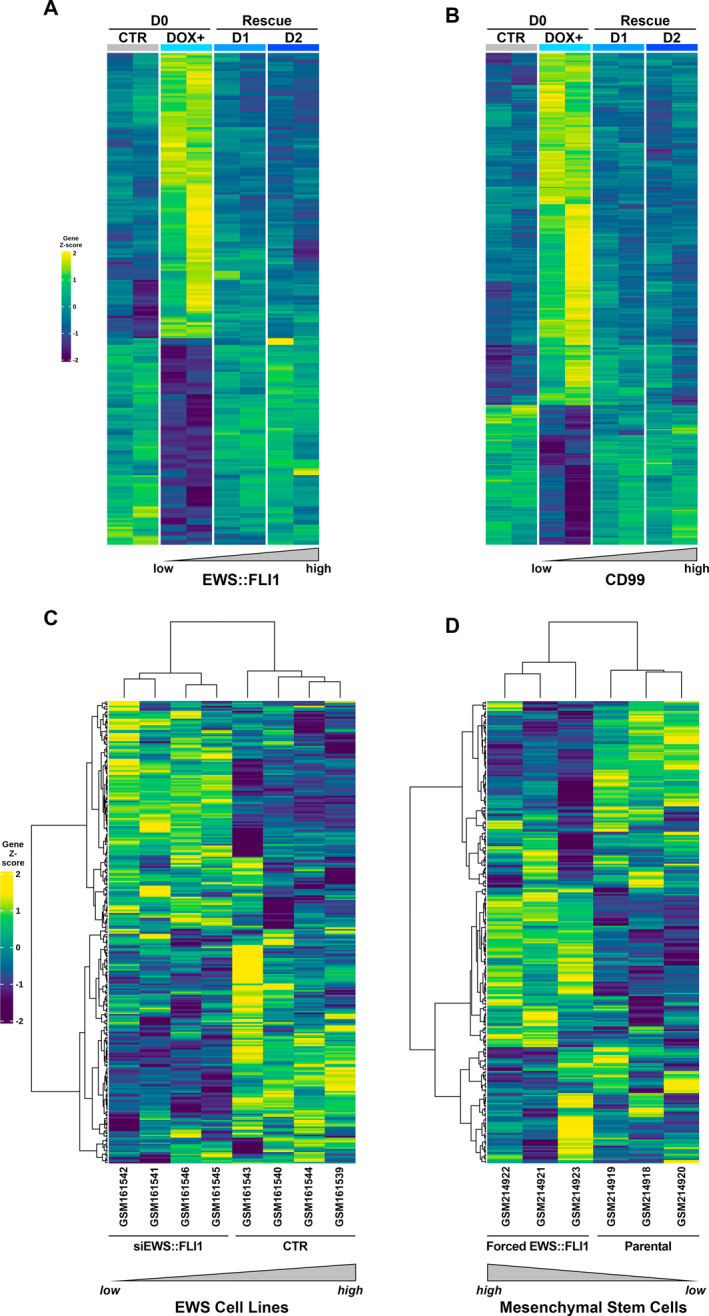
Heatmap visualization of genes whose expression changed over time in response to dynamic silencing/re‐expressing of EWS::FLI1 or CD99 and contribution of A673pTERshCD99 signature to EwS phenotype. (A) A673pTERshEWS::FLI1 cells and (B) A673pTERshCD99 cells. Control cells (CTR) were compared to doxycycline (DOX) treated cells at D0 and cells undergoing rescue at D1 and D2 with an increasing expression from low to high of EWS::FLI1 or CD99, respectively. (C) Unsupervised Hierarchical clustering using A673pTERshCD99 signature was applied to in silico datasets generated from two EWS::FLI1‐silenced EwS cell lines EW24 and SKNMC; and to (D) human bone marrow‐derived mesenchymal progenitor cells UET‐13 with ectopic expression of EWS::FLI1 fusion. The hierarchical clustering algorithm recognized two distinct clusters in both EWS::FLI1‐silenced cell lines compared to controls, and in UET‐13 with forced expression of EWS::FLI1 compared to UET‐13 parental cells. In the matrix, each row represents a gene, and each column represents a sample. The color scale illustrates the relative expression levels (z‐score) of gene across all samples: blue represents the expression level above the mean and yellow represents the expression lower than the mean.

Transcriptome profiles of A673pTERshEWS::FLI1 and A673pTERshCD99 were generated by microarray‐based gene expression technology. At D0, the silencing of EWS::FLI1 modulated the expression of 554 genes: 326 genes were upregulated and 228 were downregulated (Table S2). The silencing of CD99 induced gene expression modulation of 920 genes, 555 were upregulated and 365 genes were downregulated (Table S3), thus confirming that both molecules impacted the EwS transcriptional landscape. The analysis of genes modulated in response to dynamic silencing/re‐expressing of EWS::FLI1 or CD99, identified 221 or 427 differentially expressed genes, respectively (Figure 4A,B, and Tables S4 and S5), 92 of which were in common (Figure S1 and Tables S6).

The CD99‐associated gene signature of 427 genes was validated in the TC‐71 and IOR/BRZ EwS cell lines and CD99‐silenced variants.^9^ In particular, unsupervised HC showed that the gene signature obtained from the dynamic silencing of CD99 in A673pTERshCD99 cells was able to distinguish TC‐71 and IOR/BRZ parental cells from their CD99‐silenced variants (Figure S2). Next, to evaluate the contribution of the A673pTERshCD99 signature to EWS::FLI1‐mediated transcriptome, we used two independent, previously published datasets of the transcriptome associated with EWS::FLI1‐silencing in EwS cell lines,^16^ or with forced expression of EWS::FLI1 in mesenchymal stem cells.^13^ Applying unsupervised HC, we found that the A673pTERshCD99 signature generated in vitro in response to dynamic silencing/re‐expression of CD99, was able to distinguish EW24 and SK‐N‐MC cell lines silenced for EWS::FLI1 from control (Figure 4C). The CD99 signature also yielded a clear separation of mesenchymal stem cells overexpressing EWS::FLI1 from parental cells (Figure 4D), thus suggesting that a CD99 signature can recognize EWS::FLI driven cells.

### 3.3 Clustering of differentially expressed genes

We then employed the short time‐series expression miner (STEM) algorithm to group the differentially expressed genes identified in the A673pTERshEWS::FLI1 or A673pTERshCD99 models into time‐dependent gene clusters.

**FIGURE 5:**
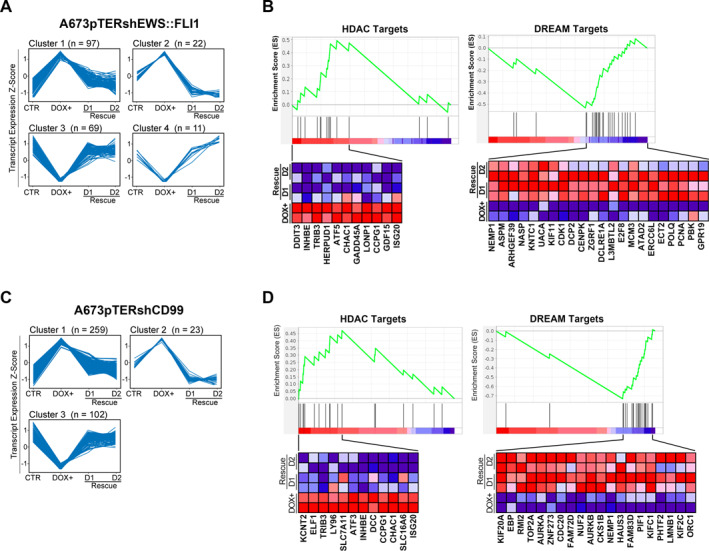
STEM algorithm and GSEA identified temporal expression patterns and functional roles into peculiar biological processes. (A) Four expression clusters were detected during silencing and re‐expression of EWS::FLI1. Clusters 1 and 2 showed genes that were upregulated when EWS::FLI1 was silenced and repressed after EWS::FLI1 rescue. Clusters 3 and 4 included genes downregulated during EWS::FLI1 silencing that became upregulated after rescue. (B) GSEA revealed a significant enrichment of up‐regulated genes in epigenetic processes and of down‐regulated genes in targets of DREAM complex. (C) Three expression clusters were detected during silencing and re‐expression of CD99. (D) GSEA revealed a significant enrichment of up‐regulated genes in epigenetic processes and of down‐regulated genes in targets of DREAM complex. Transcript expression levels are shown as transcript‐wise Z score of log_2_ expression values. For each cluster, the number of genes is shown in parentheses. The enrichment score curve was obtained using GSEA software using a continuous phenotype label and Pearson's correlation metric for ranking genes. In the enrichment plot, the *x*‐axis shows the rank order of genes from the most upregulated to the most downregulated between D0, D1 and D2. The vertical black line indicates the position of the enriched genes (Hit) comprising the gene set. The heatmaps show the genes that contribute most to the core enriched pathway, red and blue colors indicate genes up–and down‐regulated, respectively.

In A673pTERshEWS::FLI1 we detected eight significant expression profiles (Figure S3A and Table S7), which were grouped into four expression clusters. Cluster 1 (97 genes) and Cluster 2 (22 genes) included genes upregulated when EWS::FLI1 was silenced, which were downregulated as soon as the fusion oncogene was re‐expressed, while Cluster 3 (69 genes) and Cluster 4 (11 genes) contained downregulated genes in the silencing condition that became upregulated as soon as EWS::FLI1 was active again (Figure 5A). To identify classes of genes that were functionally associated with the four clusters, we performed GSEA and identified 16 top gene sets (NES >1.8, *p* < 0.01; Table S8). Genes belonging to Cluster 1 and Cluster 2 were enriched in epigenetic processes, such as histone deacetylase (HDAC) targets,^27^, ^28^ while genes of Cluster 3 were enriched in targets of the dimerization partner, RB‐like, E2F, and multi‐vulval class B (DREAM) complex, a protein complex responsible for the regulation of cell cycle‐dependent gene expression^12^, ^29^ (Figure 5B). Cluster 4 contained genes like LIPI, IGF10, and LOXHD1, which are known to be directly modulated by EWS::FLI1.^30^, ^31^, ^32^

In the A673pTERshCD99 model, seven significant expression profiles were identified (Figure S3B and Table S9). These profiles were grouped to form three expression clusters: Cluster 1 (259 genes) and Cluster 2 (23 genes) included genes upregulated in the CD99 silencing condition, but downregulated as soon as CD99 was re‐expressed; while Cluster 3 (102 genes) comprised, genes downregulated when cells are deprived of CD99 but overexpressed when the expression of the molecule was regained (Figure 5C). GSEA analysis identified 21 top gene sets (NES>1.8, *p* < 0.01; Table S10) showing that genes included in Cluster 1 and Cluster 2 also resulted enriched in histone modification^27^ via HDAC targets, while genes included in Cluster 3 were enriched in the DREAM complex targets^12^, ^29^ (Figure 5D).

GSEA confirmed the enrichment of genes belonging to Cluster 3 among the targets of EWS::FLI1 reported by Kinsey et al.,^33^ both following EWS::FLI1 (Figure S4A) or CD99 (Figure S4B) silencing, thus indicating that the gene sets associated to the two hallmarks of EwS define similar biological/molecular processes. However, the core genes that drive the enrichment score in the gene set regulated by CD99 are different from those dependent on EWS::FLI1, supporting a functional complementarity between EWS::FLI1 and CD99 in the regulation of tumor malignancy.

To corroborate this evidence, we matched the genes included in Clusters 1‐–2 of both models (genes upregulated following the EWS::FLI1 or CD99 silencing) with two previously reported meta‐analyses of EwS transcriptional profiles.^34^, ^35^ We found a strong overlap between genes included in Cluster 1‐–2 and genes up‐regulated in the EWS::FLI1 knockdown cell lines reported by Kauer et al.^34^ (*p*‐value = 3.6e‐09 in A673pTERshEWS::FLI1 and *p*‐value = 1.4e‐12 in A673pTERshCD99, hypergeometric test) and between genes belonging to Cluster 1‐–2 and genes that were down‐regulated in the “core EWS‐FLI transcriptional signature” reported by Hancock and Lessnick (*p*‐value = 4.8e‐03 in A673pTERshEWS::FLI1 and *p*‐value = 3.8e‐06 in A673pTERshCD99, hypergeometric test; Figure S5A and B).

Then, we investigated whether the genes included in Cluster 3 of both models (genes downregulated following EWS::FLI1 or CD99 silencing) which were enriched in the DREAM complex target genes pertained to transcriptomic signatures associated with specific phases of the cell cycle.^36^ We found that while genes regulated by EWS::FLI1 spanned the G1/S and G2/M phases of the cell cycle (Figure 5B), genes regulated by CD99 were more G2/M‐like (Figure 5D), with a significant overlap of 18 genes between Cluster 3 and G2/M phase specific genes identified by Dominguez et al.^36^ (*p*‐value = 2.2e‐06, hypergeometric test; Figure S5C).

### 3.4 In silico DREAM complex target genes prognostic value

**FIGURE 6:**
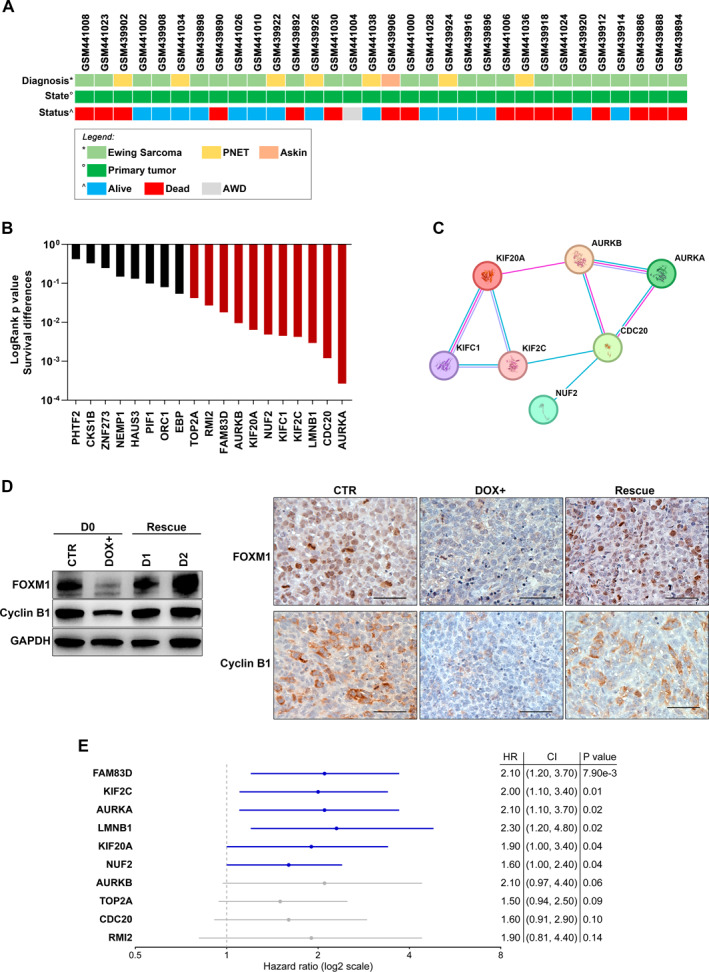
Prognostic value of DREAM target genes identified in A673pTERshCD99 model. (A) Clinical characteristics of 32 primary EwS tumor from “Mixed Ewing Sarcoma—Savola—117—MAS5.0—u133p2” dataset (GSE17618). (B) Bar graph of the log‐rank test *p* values for overall survival (OS) differences based on high versus low expression of each of the 19 CD99‐modulated genes of the core DREAM gene set. (C) Protein‐protein interactions, according to STRING database, for the genes associated with shorter OS. Only interactions supported by experimental evidence and curated database were used for this analysis. (D) Protein expression of FOXM1 and Cyclin B1 by Western Blot (left). GAPDH was used as a loading control. Representative blots from three independent experiments are shown. Immunohistochemistry staining (right) of FOXM1 and Cyclin B1 in A673pTERshCD99 xenografts (scale bar, 50 μm). (E) Multivariate Cox regression analysis identified six independent prognostic factors for EwS overall survival (HR: Hazard Ratio; CI: Confidence Interval). CI, confidence interval; HR, hazard ratio; OS, overall survival.

We validated the contribution to EwS aggressiveness of the CD99 core genes enriched in the DREAM targets by means of R2 Genomics Platform reporting microarray datasets from EwS tumors that have well‐annotated clinical information. Considering the information related to 32 primary localized EwS tumors from the dataset of Savola et al. (GSE17618^37^; Figure 6A), we found that 11 out of the 19 CD99‐modulated genes of the core DREAM gene set (one was not annotated in the Savola dataset) were associated with shorter OS when expressed at high levels (Figure 6B); Kaplan–Meier curves are shown in Figure S6. Of note, genes significantly correlating with survival were: (a) all grouped in the G2/M cell cycle phase according to Dominguez et al.^36^; (b) 9 out of 11 genes were reported to be target genes of the Forkhead box M1 (FOXM1) transcription factor in low‐ or high‐throughput transcription factor functional studies from the CHEA Transcription Factor Targets dataset (https://maayanlab.cloud/Harmonizome/gene_set/FOXM1/CHEA+Transcription+Factor+Targets); and (c) 7 of these genes resulted functionally interconnected according to the STRING database^38^ (Figure 6C), considering only interactions supported by experimental evidence and curated database.

Classical regulators of gene expression during cell cycle include the transcription factors E2F1 and FOXM1 that are required for the temporal control of gene expression in G1/S and G2/M phases, respectively.^36^, ^39^ Accordingly, we found that loss of CD99 led to the reduction of FOXM1 protein levels and some FOXM1 targets, such as cyclin B1, while its re‐expression also rescued the expression of FOXM1 and associated targets (Figure 6D). Multivariate Cox regression analysis confirmed the clinical relevance of six genes (Figure 6E), all of them reported to be targets of FOXM1. In A673pTERshCD99 the mRNA expression of FOXM1 and of *FAM83D*, *KIF2C*, *AURKA*, *LMNB1*, *KIF2*0A, *and NUF2* dynamically followed that of CD99 (Figure S7). As validation, we used three other EwS experimental models (named as BRZ‐CD99‐shRNA, CAR‐CD99‐shRNA, and TC‐CD99‐shRNA) that were stable silenced for CD99 expression.^9^, ^17^ By using RT‐qPCR, we tested the relative expression of *FOXM1* and the six targets and demonstrated their significant downregulation in cells deprived of CD99 (Figure S8).The clinical relevance of this core‐set of genes was cross‐validated in an additional dataset (GSE63157^25^) that includes 85 cases of EwS without indication of their origin (i.e., primary, recurrence, or metastasis). Multivariate analysis confirmed the prognostic value of *FAM83D*, *KIF2C*, and *AURKA*, further supporting the clinical relevance of genes that are regulated by *FOXM1* and involved in the G2/M and late M‐phase of cell cycle (Figure S9A and B).

## 4 DISCUSSION

We used EwS cell lines stably transfected with an inducible shRNA system targeting the two hallmarks of EwS, EWS::FLI1, and CD99, to obtain a mechanistic picture of the causal relations between the two genes. Classical approaches for elucidating gene function usually look at upstream regulators and down‐stream targets within a pathway, thus missing possible interplays with other molecular mediators. The analysis of time‐related transcriptome responses to perturbed experimental systems may provide a more precise description of the evolution of molecular networks in response to various perturbations.

EwS cell proliferation in vitro and xenograft tumor growth were inhibited when either CD99 or EWS::FLI1 was silenced with DOX, and took off again once DOX was withdrawn, illustrating the independent contribution of both molecules to EwS growth. Silencing of CD99 provided novel information on the genes modulated by CD99, their interaction with the genes modulated by EWS::FLI1, and their relevance for the maintenance of EwS malignancy. Furthermore, our data supported the concept that EWS::FLI1 regulates complex networks, including those of genes connected to the epigenetic regulation of gene expression and tumor cell proliferation.^28^, ^30^

The results of inducible CD99 silencing reported here are in agreement with those previously obtained with anti‐CD99 agonistic antibodies (Abs). These Abs efficiently deliver a cell death message to EwS cells, but they do not affect the viability or the differentiation of normal human hematopoietic or mesenchymal stem cells, which also express high levels of CD99^10^, ^40^ in the absence of EWS::FLI1. In contrast, when normal mesenchymal stem cells were transfected with EWS::FLI1, their susceptibility to CD99 ligation increased, thus supporting the idea that CD99‐mediated delivery of intrinsic cell death signals is favored by the presence of the EWS::FLI1 oncogene.^10^ Loss of CD99 either through antibodies or siRNA interference approaches has been reported to repress the expression of the genes regulated by GLI‐1,^11^ a direct transcriptional target of EWS::FLI1 that has been found to be essential in EWS::FLI1–induced signaling and required to sustain the malignant phenotype of EwS cells.^41^ Thus, in the absence of CD99, EwS cells proliferate and migrate less, are more prone to dye and/or differentiate and express lower levels of some targets of EWS::FLI1, even in the presence of an active oncogenic chimera.

Through the analysis of the transcriptome associated with EWS::FLI1 or CD99 expression, we demonstrated an overlap in the biological processes that are regulated by the two molecules. In particular, genes overexpressed when either hallmark was silenced were found to be enriched in targets of HDAC, whereas, genes repressed in the absence of the molecules were enriched in targets of the DREAM complex, thus indicating that both EWS::FLI1 and CD99 suppress gene expression through histone modifications and sustain genes governing cell proliferation. Interestingly, only a modest overlap was found between the genes of these two major processes specifically associated with either EWS::FLI1 or CD99.

The main function of the DREAM complex is to repress G1/S and G2/M gene expression during quiescence (G0).^12^ Our data suggest that the targets of the DREAM complex regulated by EWS::FLI1 are distributed throughout the different phases of the cell cycle, whereas the genes regulated by CD99 are more specific of G2/M.^36^ This is consistent with our previous observation that EwS cells with a stable silencing of CD99 are arrested in the G2/M phase.^9^

Progression through the cell cycle requires the periodic expression of phase‐specific gene clusters.^12^ Regulatory mechanisms are highly conserved and include the transcription factors E2F1 and FOXM1, which are classically described as temporal controllers of gene expression in G1/S and G2/M phases of cell cycle, respectively.^36^, ^39^ Here, we showed that the expression of *FOXM1* and its targets went in parallel with that of CD99 in four different cell EwS cell lines. It is possible that CD99 regulates the expression of FOXM1 through GLI1. FOXM1 was determined to be a transcriptional target of GLI1 and functions downstream Hedgehog/GLI1 signaling.^42^ We have recently demonstrated silencing of CD99 in EwS cells is sufficient to increase the expression of zyxin, a zinc‐finger protein that was shown to play a role in transducing stimuli from the cell membrane to the nucleus expression. Deprivation of CD99 induces recruitment of zyxin to the nucleus, where it can interact with the transcription factor GLI1 inhibiting its activity.^11^

FOXM1 is expressed at robust levels in a variety of EwS tumor specimens and in EwS cell lines.^43^ In particular, Christensen et al.^43^ demonstrated that EWS::FLI1 increased the expression levels of FOXM1 in four different EwS cell lines, but they did not find evidence that FOXM1 was directly targeted by EWS::FLI1. Considering that the forced expression of EWS::FLI1 also increases the expression of CD99,^7^, ^9^ our data indicate that CD99 may be the missing link.

Among the CD99‐regulated genes that were found to be enriched as targets of the DREAM complex, all those associated with a bad clinical prognosis are targets of FOXM1. Three of them (*FAM83D*, *KIF2C*, and *AURKA*) were validated in silico in two different cohorts of patients. Thus, our data uncovered a transcriptional G2/M‐related gene cluster regulated by FOXM1 that is important for EwS progression and may be considered for therapeutic intervention. Upregulation of FOXM1 has been described in many cancers, acting as a master transcriptional regulator that promotes tumor progression, metastasis, and chemoresistance.^44^ Multiple attempts were made to develop FOXM1 inhibitors, including proteasome inhibitors, thiazole antibiotics and small molecule inhibitors inducing FOXM1 degradation.^45^ In EwS, treatment with thiostrepton, a proteasomal inhibitor reported to physically interact with FOXM1, hindering its ability to bind to its target promoters^46^ was demonstrated to effectively inhibit tumor growth in mouse xenografts.^47^ Despite encouraging preclinical data, clinical FOXM1 targeting is still a challenging task. The evidence provided here that FOXM1 may be inhibited through the targeting of a cell surface molecule, like CD99, may offer a promising alternative for those tumors that overexpress CD99 (i.e., EwS, glioma, melanoma, and acute myeloid leukemia)^48^, ^49^

In conclusion, through the dynamic modulation of CD99 in EwS cells, we highlighted the fundamental contribution of this molecule to the malignancy of EwS. Our data indicate that, after the oncogenic fusion of EWS and FLI1, the genetic landscape of EwS is also regulated by CD99 whose expression is favored by, but also independent of EWS::FLI1. CD99 was not included in the dynamics of the EWS::FLI1 signature, and only a partial overlap is observed among the genes associated with changes in the expression of either EWS::FLI1 or CD99. However, the two molecules converged on similar biological pathways, thus supporting the idea of cooperation rather than dependence. It must be considered that human mesenchymal stem cells, the putative cell of origin of EwS, normally express high levels of CD99. Therefore, CD99 may be already expressed before the oncogenic EWS::FLI1 fusion, which might provide only a minor contribution to CD99 regulation, while the transformed cell may gain advantage from the cooperative functions of the two genes. The involvement of CD99 in the regulation of tumor growth through a specific modulation of FOXM1‐regulated genes involved in G2/M phase offers a core‐set of genes with prognostic value and opens new therapeutic perspectives.

## AUTHOR CONTRIBUTIONS

**Michela Pasello**: Conceptualization; data curation; writing – original draft; writing – review and editing; analysis and interpretation of data; graphed the data. **Maria Antonella Laginestra**: Conceptualization; bioinformatics analysis; writing – original draft; writing – review and editing; analysis and interpretation of data; graphed the data. **Maria Cristina Manara**: Data curation; review and editing; data acquisition. **Lorena Landuzzi**: In vivo data curation; writing – review and editing; in vivo data acquisition; analysis and interpretation of data. **Francesca Ruzzi**: In vivo data curation; review and editing; in vivo data acquisition; analysis and interpretation of data. **Margherita Maioli**: Data curation; review and editing; data acquisition. **Evelin Pellegrini**: Data curation; review and editing; data acquisition. **Alessandra De Feo**: Data curation; review and editing; data acquisition. **Pier‐Luigi Lollini**: Resources; writing – review and editing. **Katia Scotlandi**: Conceptualization; resources; data curation; supervision; writing – original draft; writing – review and editing.

## CONFLICT OF INTEREST STATEMENT

No potential conflicts of interest were disclosed by the other authors.

## ETHICS STATEMENT

All animal procedures were done in accordance with European directive 2010/63/UE and Italian Law (DL 26/2014) and following ARRIVE guidelines.^50^ Experimental protocols were reviewed and approved by the institutional animal care and use committee (“Comitato per il Benessere Animale”) of the University of Bologna and by the Italian Ministry of Health with letters 208/2017‐PR and 1051/2020‐PR.

## Supporting information

Supporting Information S1

Supporting Information S2

## Data Availability

The microarray gene expression data generated in this study was carried out according to MIAME (Minimum Information About a Microarray Experiment) guidelines and has been deposited in the National Center for Biotechnology Information Gene Expression Omnibus (GEO) and are accessible through the GEO Series accession number GSE256247. Other data are available within the article and its Supplementary Data files or from the corresponding author upon request.

## References

[1] DuBois, Steven G. , Mark D. Krailo , Julia Glade‐Bender , Allen Buxton , Nadia Laack , R. Lor Randall , Helen X. Chen , et al. 2023. “Randomized Phase III Trial of Ganitumab with Interval‐Compressed Chemotherapy for Patients with Newly Diagnosed Metastatic Ewing Sarcoma: A Report from the Children's Oncology Group.” Journal of Clinical Oncology 41(11): 2098–2107. 10.1200/jco.22.01815. 36669140 PMC10082251

[2] Koch, Raphael , Hans Gelderblom , Lianne Haveman , Benedicte Brichard , Heribert Jürgens , Sona Cyprova , Henk van den Berg , et al. 2022. “High‐Dose Treosulfan and Melphalan as Consolidation Therapy versus Standard Therapy for High‐Risk (Metastatic) Ewing Sarcoma.” Journal of Clinical Oncology 40(21): 2307–2320. 10.1200/jco.21.01942. 35427190

[3] Attia, Steven , Vanessa Bolejack , Kristen N. Ganjoo , Suzanne George , Mark Agulnik , Daniel Rushing , Elizabeth T. Loggers , et al. 2023. “A Phase II Trial of Regorafenib in Patients with Advanced Ewing Sarcoma and Related Tumors of Soft Tissue and Bone: SARC024 Trial Results.” Cancer Medicine 12(2): 1532–1539. 10.1002/cam4.5044. 35950293 PMC9883574

[4] Crompton, Brian D. , Chip Stewart , Amaro Taylor‐Weiner , Gabriela Alexe , Kyle C. Kurek , Monica L. Calicchio , Adam Kiezun , et al. 2014. “The Genomic Landscape of Pediatric Ewing Sarcoma.” Cancer Discovery 4(11): 1326–1341. 10.1158/2159-8290.cd-13-1037. 25186949

[5] Tirode, Franck , Didier Surdez , Xiaotu Ma , Matthew Parker , Marie Cécile Le Deley , Armita Bahrami , Zhaojie Zhang , et al. 2014. “Genomic Landscape of Ewing Sarcoma Defines an Aggressive Subtype with Co‐association of STAG2 and TP53 Mutations.” Cancer Discovery 4(11): 1342–1353. 10.1158/2159-8290.cd-14-0622. 25223734 PMC4264969

[6] Grünewald, Thomas G. P. , Florencia Cidre‐Aranaz , Didier Surdez , Eleni M. Tomazou , Enrique de Álava , Heinrich Kovar , Poul H. Sorensen , Olivier Delattre , and Uta Dirksen . 2018. “Ewing Sarcoma.” Nature Reviews Disease Primers 4(1): 5. 10.1038/s41572-018-0003-x. 29977059

[7] Franzetti, G. A. , K. Laud‐Duval , W. van der Ent , A. Brisac , M. Irondelle , S. Aubert , U. Dirksen , et al. 2017. “Cell‐to‐cell Heterogeneity of EWSR1‐FLI1 Activity Determines Proliferation/migration Choices in Ewing Sarcoma Cells.” Oncogene 36(25): 3505–3514. 10.1038/onc.2016.498. 28135250 PMC5541267

[8] Chaturvedi, A. , L. M. Hoffman , A. L. Welm , S. L. Lessnick , and M. C. Beckerle . 2012. “The EWS/FLI Oncogene Drives Changes in Cellular Morphology, Adhesion, and Migration in Ewing Sarcoma.” Genes Cancer 3(2): 102–116. 10.1177/1947601912457024. 23050043 PMC3463921

[9] Rocchi, Anna , Maria Cristina Manara , Marika Sciandra , Diana Zambelli , Filippo Nardi , Giordano Nicoletti , Cecilia Garofalo , et al. 2010. “CD99 Inhibits Neural Differentiation of Human Ewing Sarcoma Cells and Thereby Contributes to Oncogenesis.” Journal of Clinical Investigation 120(3): 668–680. 10.1172/jci36667. 20197622 PMC2827943

[10] Guerzoni, Clara , Valentina Fiori , Mario Terracciano , Maria Cristina Manara , Diego Moricoli , Michela Pasello , Marika Sciandra , et al. 2015. “CD99 Triggering in Ewing Sarcoma Delivers a Lethal Signal through P53 Pathway Reactivation and Cooperates with Doxorubicin.” Clinical Cancer Research 21(1): 146–156. 10.1158/1078-0432.ccr-14-0492. 25501132

[11] Balestra, Tommaso , Maria Cristina Manara , Maria Antonella Laginestra , Michela Pasello , Alessandra De Feo , Cristian Bassi , Clara Guerzoni , et al. 2022. “Targeting CD99 Compromises the Oncogenic Effects of the Chimera EWS‐FLI1 by Inducing Reexpression of Zyxin and Inhibition of GLI1 Activity.” Molecular Cancer Therapeutics 21(1): 58–69. 10.1158/1535-7163.mct-21-0189. 34667115

[12] Sadasivam, Subhashini , and James A. DeCaprio . 2013. “The DREAM Complex: Master Coordinator of Cell Cycle‐dependent Gene Expression.” Nature Reviews Cancer 13(8): 585–595. 10.1038/nrc3556. 23842645 PMC3986830

[13] Miyagawa, Yoshitaka , Hajime Okita , Hideki Nakaijima , Yasuomi Horiuchi , Ban Sato , Tomoko Taguchi , Masashi Toyoda , et al. 2008. “Inducible Expression of Chimeric EWS/ETS Proteins Confers Ewing's Family Tumor‐like Phenotypes to Human Mesenchymal Progenitor Cells.” Molecular and Cellular Biology 28(7): 2125–2137. 10.1128/mcb.00740-07. 18212050 PMC2268432

[14] Hu‐Lieskovan, Siwen , Jingsong Zhang , Lingtao Wu , Hiroyuki Shimada , Deborah E. Schofield , and Timothy J. Triche . 2005. “EWS‐FLI1 Fusion Protein Up‐Regulates Critical Genes in Neural Crest Development and Is Responsible for the Observed Phenotype of Ewing's Family of Tumors.” Cancer Research 65(11): 4633–4644. 10.1158/0008-5472.can-04-2857. 15930281

[15] van de Wetering, Marc , Elena Sancho , Cornelis Verweij , Wim de Lau , Irma Oving , Adam Hurlstone , Karin van der Horn , et al. 2002. “The Beta‐catenin/TCF‐4 Complex Imposes a Crypt Progenitor Phenotype on Colorectal Cancer Cells.” Cell 111(2): 241–250. 10.1016/s0092-8674(02)01014-0. 12408868

[16] Tirode, Franck , Karine Laud‐Duval , Alexandre Prieur , Bruno Delorme , Pierre Charbord , and Olivier Delattre . 2007. “Mesenchymal Stem Cell Features of Ewing Tumors.” Cancer Cell 11(5): 421–429. 10.1016/j.ccr.2007.02.027. 17482132

[17] Ventura, S. , D. N. T. Aryee , F. Felicetti , A. De Feo , C. Mancarella , M. C. Manara , P. Picci , et al. 2016. “CD99 Regulates Neural Differentiation of Ewing Sarcoma Cells through miR‐34a‐Notch‐Mediated Control of NF‐kappaB Signaling.” Oncogene 35(30): 3944–3954. 10.1038/onc.2015.463. 26616853 PMC4967355

[18] Livak, Kenneth J. , and Thomas D. Schmittgen . 2001. “Analysis of Relative Gene Expression Data Using Real‐Time Quantitative PCR and the 2(‐Delta Delta C(T)) Method.” Methods 25(4): 402–408. 10.1006/meth.2001.1262. 11846609

[19] Redelsperger, I. M. , T. Taldone , E. R. Riedel , M. L. Lepherd , N. S. Lipman , and F. R. Wolf . 2016. “Stability of Doxycycline in Feed and Water and Minimal Effective Doses in Tetracycline‐Inducible Systems.” Journal of the American Association for Laboratory Animal Science 55: 467–474. 27423155 PMC4943619

[20] Ritchie, Matthew E. , Belinda Phipson , Di Wu , Yifang Hu , Charity W. Law , Wei Shi , and Gordon K. Smyth . 2015. “Limma Powers Differential Expression Analyses for RNA‐Sequencing and Microarray Studies.” Nucleic Acids Research 43(7): e47. 10.1093/nar/gkv007. 25605792 PMC4402510

[21] Gu, Zuguang , Roland Eils , and Matthias Schlesner . 2016. “Complex Heatmaps Reveal Patterns and Correlations in Multidimensional Genomic Data.” Bioinformatics 32(18): 2847–2849. 10.1093/bioinformatics/btw313. 27207943

[22] Ernst, J. , G. J. Nau , and Z. Bar‐Joseph . 2005. “Clustering Short Time Series Gene Expression Data.” Bioinformatics 21(Suppl 1): i159–i168. 10.1093/bioinformatics/bti1022. 15961453

[23] Wickam, H. 2016. ggplot2: Elegant Graphics for Data Analysised.

[24] Subramanian, Aravind , Pablo Tamayo , Vamsi K. Mootha , Sayan Mukherjee , Benjamin L. Ebert , Michael A. Gillette , Amanda Paulovich , et al. 2005. “Gene Set Enrichment Analysis: a Knowledge‐Based Approach for Interpreting Genome‐wide Expression Profiles.” Proceedings of the National Academy of Sciences of the USA 102(43): 15545–15550. 10.1073/pnas.0506580102. 16199517 PMC1239896

[25] Selvanathan, Saravana P. , Garrett T. Graham , Hayriye V. Erkizan , Uta Dirksen , Thanemozhi G. Natarajan , Aleksandra Dakic , Songtao Yu , et al. 2015. “Oncogenic Fusion Protein EWS‐FLI1 Is a Network Hub that Regulates Alternative Splicing.” Proceedings of the National Academy of Sciences of the USA 112(11): E1307–E1316. 10.1073/pnas.1500536112. 25737553 PMC4371969

[26] Bradburn, M. J. , T. G. Clark , S. B. Love , and D. G. Altman . 2003. “Survival Analysis Part III: Multivariate Data Analysis – Choosing a Model and Assessing its Adequacy and Fit.” British Journal of Cancer 89(4): 605–611. 10.1038/sj.bjc.6601120. 12915864 PMC2376927

[27] Schmidt, Oxana , Nadja Nehls , Carolin Prexler , Kristina von Heyking , Tanja Groll , Katharina Pardon , Heathcliff D. Garcia , et al. 2021. “Class I Histone Deacetylases (HDAC) Critically Contribute to Ewing Sarcoma Pathogenesis.” Journal of Experimental & Clinical Cancer Research 40(1): 322. 10.1186/s13046-021-02125-z. 34654445 PMC8518288

[28] Heller, Gerwin , Wolfgang M. Schmidt , Barbara Ziegler , Sonja Holzer , Leonhard Müllauer , Martin Bilban , Christoph C. Zielinski , Johannes Drach , and Sabine Zöchbauer‐Müller . 2008. “Genome‐wide Transcriptional Response to 5‐Aza‐2'‐Deoxycytidine and Trichostatin a in Multiple Myeloma Cells.” Cancer Research 68(1): 44–54. 10.1158/0008-5472.can-07-2531. 18172295

[29] Fischer, Martin , Patrick Grossmann , Megha Padi , and James A. DeCaprio . 2016. “Integration of TP53, DREAM, MMB‐FOXM1 and RB‐E2f Target Gene Analyses Identifies Cell Cycle Gene Regulatory Networks.” Nucleic Acids Research 44(13): 6070–6086. 10.1093/nar/gkw523. 27280975 PMC4994865

[30] Aynaud, M.‐Ming , Olivier Mirabeau , Nadege Gruel , Sandrine Grossetête , Valentina Boeva , Simon Durand , Didier Surdez , et al. 2020. “Transcriptional Programs Define Intratumoral Heterogeneity of Ewing Sarcoma at Single‐Cell Resolution.” Cell Reports 30(6): 1767–1779.e6. 10.1016/j.celrep.2020.01.049. 32049009

[31] Deng, Qu , Ramakrishnan Natesan , Florencia Cidre‐Aranaz , Shehbeel Arif , Ying Liu , Reyaz ur Rasool , Pei Wang , et al. 2022. “Oncofusion‐driven De Novo Enhancer Assembly Promotes Malignancy in Ewing Sarcoma via Aberrant Expression of the Stereociliary Protein LOXHD1.” Cell Reports 39(11): 110971. 10.1016/j.celrep.2022.110971. 35705030 PMC9716578

[32] Showpnil, Iftekhar A. , Julia Selich‐Anderson , Cenny Taslim , Megann A. Boone , Jesse C. Crow , Emily R. Theisen , and Stephen L. Lessnick . 2022. “EWS/FLI Mediated Reprogramming of 3D Chromatin Promotes an Altered Transcriptional State in Ewing Sarcoma.” Nucleic Acids Research 50(17): 9814–9837. 10.1093/nar/gkac747. 36124657 PMC9508825

[33] Kinsey, Michelle , Richard Smith , and Stephen L. Lessnick . 2006. “NR0B1 Is Required for the Oncogenic Phenotype Mediated by EWS/FLI in Ewing's Sarcoma.” Molecular Cancer Research 4(11): 851–859. 10.1158/1541-7786.mcr-06-0090. 17114343

[34] Kauer, Maximilian , Jozef Ban , Reinhard Kofler , Bob Walker , Sean Davis , Paul Meltzer , and Heinrich Kovar . 2009. “A Molecular Function Map of Ewing's Sarcoma.” PLoS One 4: e5415. 10.1371/journal.pone.0005415. 19404404 PMC2671847

[35] Hancock, Jeffrey D. , and Stephen L. Lessnick . 2008. “A Transcriptional Profiling Meta‐Analysis Reveals a Core EWS‐FLI Gene Expression Signature.” Cell Cycle 7(2): 250–256. 10.4161/cc.7.2.5229. 18256529

[36] Dominguez, Daniel , Yi‐Hsuan Tsai , Nicholas Gomez , Deepak Kumar Jha , Ian Davis , and Zefeng Wang . 2016. “A High‐Resolution Transcriptome Map of Cell Cycle Reveals Novel Connections between Periodic Genes and Cancer.” Cell Research 26(8): 946–962. 10.1038/cr.2016.84. 27364684 PMC4973334

[37] Savola, Suvi , Arto Klami , Samuel Myllykangas , Cristina Manara , Katia Scotlandi , Piero Picci , Sakari Knuutila , and Jukka Vakkila . 2011. “High Expression of Complement Component 5 (C5) at Tumor Site Associates with Superior Survival in Ewing's Sarcoma Family of Tumour Patients.” ISRN Oncol 2011: 168712. 10.5402/2011/168712. 22084725 PMC3196920

[38] Szklarczyk, Damian , Rebecca Kirsch , Mikaela Koutrouli , Katerina Nastou , Farrokh Mehryary , Radja Hachilif , Annika L. Gable , et al. 2023. “The STRING Database in 2023: Protein‐Protein Association Networks and Functional Enrichment Analyses for Any Sequenced Genome of Interest.” Nucleic Acids Research 51(D1): D638–D646. 10.1093/nar/gkac1000. 36370105 PMC9825434

[39] Grant, Gavin D. , Lionel Brooks, 3rd , Xiaoyang Zhang , J. Matthew Mahoney , Viktor Martyanov , Tammara A. Wood , Gavin Sherlock , Chao Cheng , and Michael L. Whitfield . 2013. “Identification of Cell Cycle‐Regulated Genes Periodically Expressed in U2OS Cells and Their Regulation by FOXM1 and E2F Transcription Factors.” Molecular Biology of the Cell 24(23): 3634–3650. 10.1091/mbc.e13-05-0264. 24109597 PMC3842991

[40] Scotlandi, Katia , Stefania Perdichizzi , Ghislaine Bernard , Giordano Nicoletti , Patrizia Nanni , P.‐Luigi Lollini , Antonio Curti , et al. 2006. “Targeting CD99 in Association with Doxorubicin: an Effective Combined Treatment for Ewing's Sarcoma.” European Journal of Cancer 42(1): 91–96. 10.1016/j.ejca.2005.09.015. 16326096

[41] Beauchamp, Elspeth , Gulay Bulut , Ogan Abaan , Kevin Chen , Akil Merchant , William Matsui , Yoshimi Endo , Jeffrey S. Rubin , Jeffrey Toretsky , and Aykut Üren . 2009. “GLI1 Is a Direct Transcriptional Target of EWS‐FLI1 Oncoprotein.” Journal of Biological Chemistry 284(14): 9074–9082. 10.1074/jbc.m806233200. 19189974 PMC2666556

[42] Teh, M. T. , S. T. Wong , G. W. Neill , L. R. Ghali , M. P. Philpott , and A. G. Quinn . 2002. “FOXM1 Is a Downstream Target of Gli1 in Basal Cell Carcinomas.” Cancer Research 62: 4773–4780. 12183437

[43] Christensen, Laura , Jay Joo , Sean Lee , Daniel Wai , Timothy J. Triche , and William A. May . 2013. “FOXM1 Is an Oncogenic Mediator in Ewing Sarcoma.” PLoS One 8(1): e54556. 10.1371/journal.pone.0054556. 23365673 PMC3554707

[44] Khan, Md Arafat , Parvez Khan , Aatiya Ahmad , Mahek Fatima , and Mohd Wasim Nasser . 2023. “FOXM1: A Small Fox that Makes More Tracks for Cancer Progression and Metastasis.” Seminars in Cancer Biology 92: 1–15. 10.1016/j.semcancer.2023.03.007. 36958703 PMC10199453

[45] Raghuwanshi, Sanjeev , and Andrei L. Gartel . 2023. “Small‐molecule Inhibitors Targeting FOXM1: Current Challenges and Future Perspectives in Cancer Treatments.” Biochimica et Biophysica Acta (BBA) ‐ Reviews on Cancer 1878(6): 189015. 10.1016/j.bbcan.2023.189015. 37913940

[46] Hegde, Nagaratna S. , Deborah A. Sanders , Raphaël Rodriguez , and Shankar Balasubramanian . 2011. “The Transcription Factor FOXM1 Is a Cellular Target of the Natural Product Thiostrepton.” Nature Chemistry 3(9): 725–731. 10.1038/nchem.1114. 21860463

[47] Sengupta, Aniruddha , Mahbubur Rahman , Silvia Mateo‐Lozano , Oscar M. Tirado , and Vicente Notario . 2013. “The Dual Inhibitory Effect of Thiostrepton on FoxM1 and EWS/FLI1 Provides a Novel Therapeutic Option for Ewing's Sarcoma.” International Journal of Oncology 43(3): 803–812. 10.3892/ijo.2013.2016. 23857410 PMC3787886

[48] Pasello, Michela , Maria Cristina Manara , and Katia Scotlandi . 2018. “CD99 at the Crossroads of Physiology and Pathology.” Journal of cell Communication and Signaling 12(1): 55–68. 10.1007/s12079-017-0445-z. 29305692 PMC5842202

[49] Shang, Erfei , Shanyue Sun , Ruolan Zhang , Zehui Cao , Qingwang Chen , Leming Shi , Jinsong Wu , Shuai Wu , Yingchao Liu , and Yuanting Zheng . 2023. “Overexpression of CD99 Is Associated with Tumor Adaptiveness and Indicates the Tumor Recurrence and Therapeutic Responses in Gliomas.” Transl Oncol 37: 101759. 10.1016/j.tranon.2023.101759. 37579711 PMC10440586

[50] Percie du Sert, Nathalie , Viki Hurst , Amrita Ahluwalia , Sabina Alam , Marc T. Avey , Monya Baker , William J. Browne , et al. 2020. “The ARRIVE Guidelines 2.0: Updated Guidelines for Reporting Animal Research.” PLoS Biology 18(7): e3000410. 10.1371/journal.pbio.3000410. 32663219 PMC7360023

