# Supplementary material for: CD99 contributes to the EWS::FLI1 transcriptome by specifically affecting FOXM1‐targets involved in the G2/M cell cycle phase, thus influencing the Ewing sarcoma genetic landscape
Source: J Cell Commun Signal. 2024 Aug 2;18(3):e12047. doi: 10.1002/ccs3.12047 (PMC11544636; doi:10.1002/ccs3.12047)
Supplement: Supplementary file 1 — Supporting Information S1 [file CCS3-18-e12047-s002.pdf]

SUPPLEMENTARY DATA

CD99 contributes to the EWS::FLI1 transcriptome by specifically affecting FOXM1-targets involved in the G2/M cell cycle phase, thus influencing the Ewing sarcoma genetic landscape.

Michela Pasello[1*], Maria Antonella Laginestra[1*], Maria Cristina Manara[1], Lorena Landuzzi[1], Francesca Ruzzi[2], Margherita Maioli[3], Evelin Pellegrini[1], Alessandra De Feo[1], Pier-Luigi Lollini [2], and Katia Scotlandi[1#].

[1]*Laboratory of Experimental Oncology, IRCCS Istituto Ortopedico Rizzoli, Bologna, Italy.*
[2]*Laboratory of Immunology and Biology of Metastasis, Department of Medical and Surgical Sciences (DIMEC), University of Bologna, Bologna, Italy.*
[3]*Department of Pathology, IRCCS Istituto Ortopedico Rizzoli, Bologna, Italy.*

**These authors contributed equally.*

#Correspondence:

Katia Scotlandi: Laboratory of Experimental Oncology, IRCCS Istituto Ortopedico Rizzoli, Via di Barbiano 1/10, 40136 Bologna, Italy. Telephone: (+39)-051-6366760; e-mail: katia.scotlandi@ior.it.

Supplementary Figures

Supplementary Figure S1

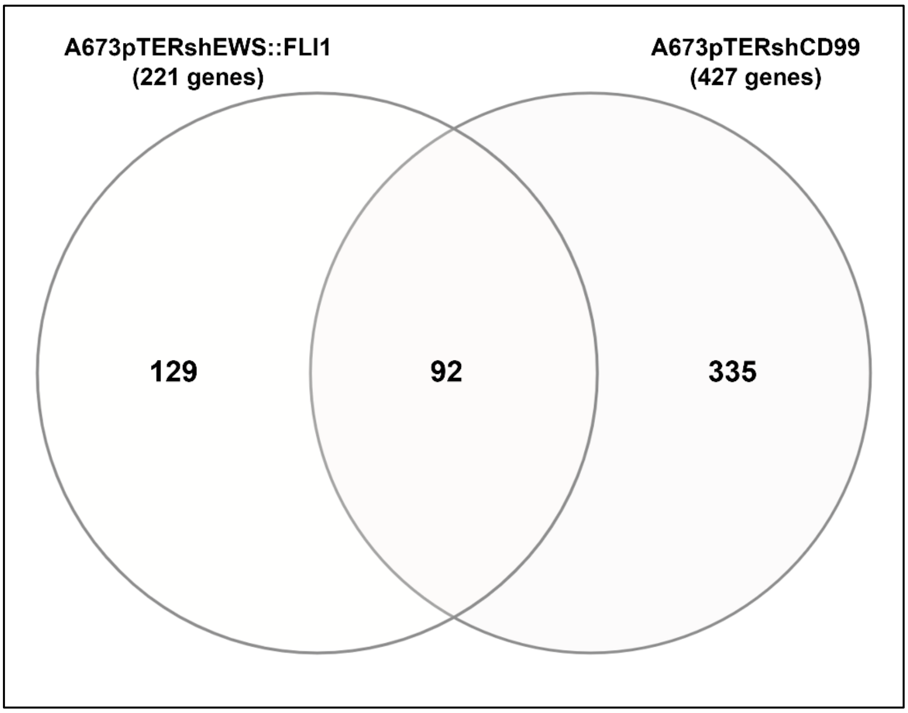

Supplementary Figure S1: Venn diagram showed the 92 shared genes between the identified A673pTERshEWS::FLI1 and A673pTERshCD99 dynamic signatures. The common genes are reported in Supplementary Table S6

Supplementary Figure S2

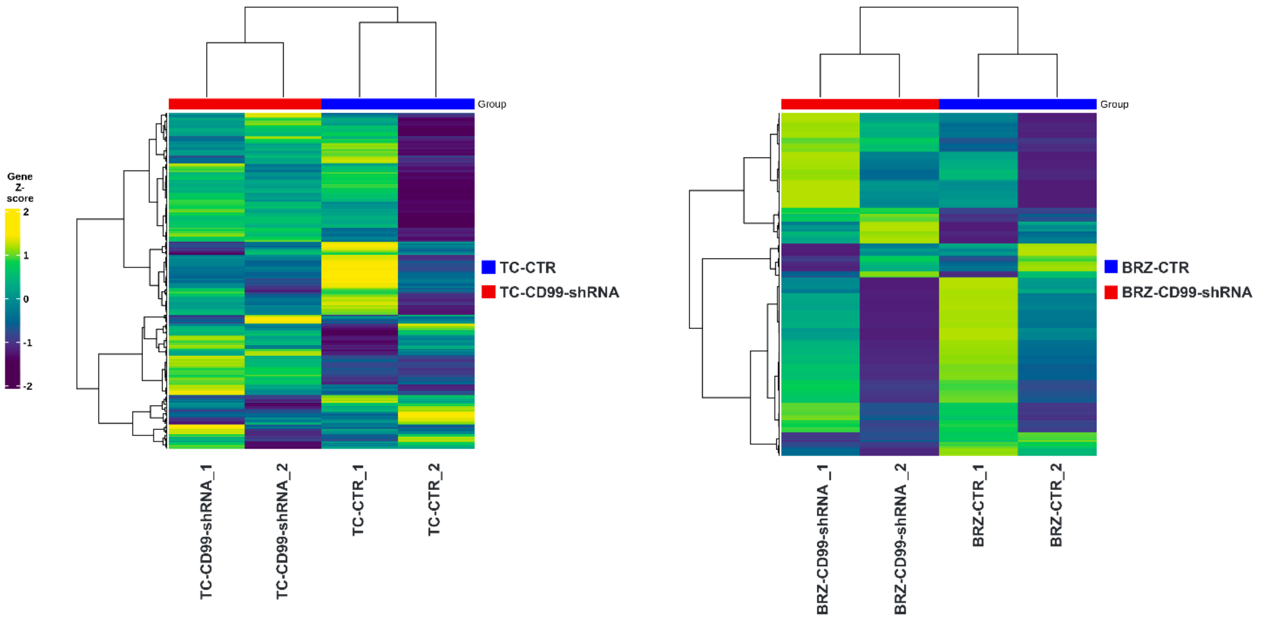

Supplementary Figure S2: Unsupervised Hierarchical clustering using A673pTERshCD99 signature was applied to TC-71 and IOR/BRZ CD99 silenced models (GSE10993) The hierarchical clustering algorithm recognized two distinct clusters in both TC-71 and IOR/BRZ-silenced cell lines compared to controls. In the matrix, each row represents a gene, and each column represents a sample. The color scale illustrates the relative expression levels (z-score) of gene across all samples: blue represents the expression level above the mean and yellow represents the expression lower than the mean.

Supplementary Figure S3

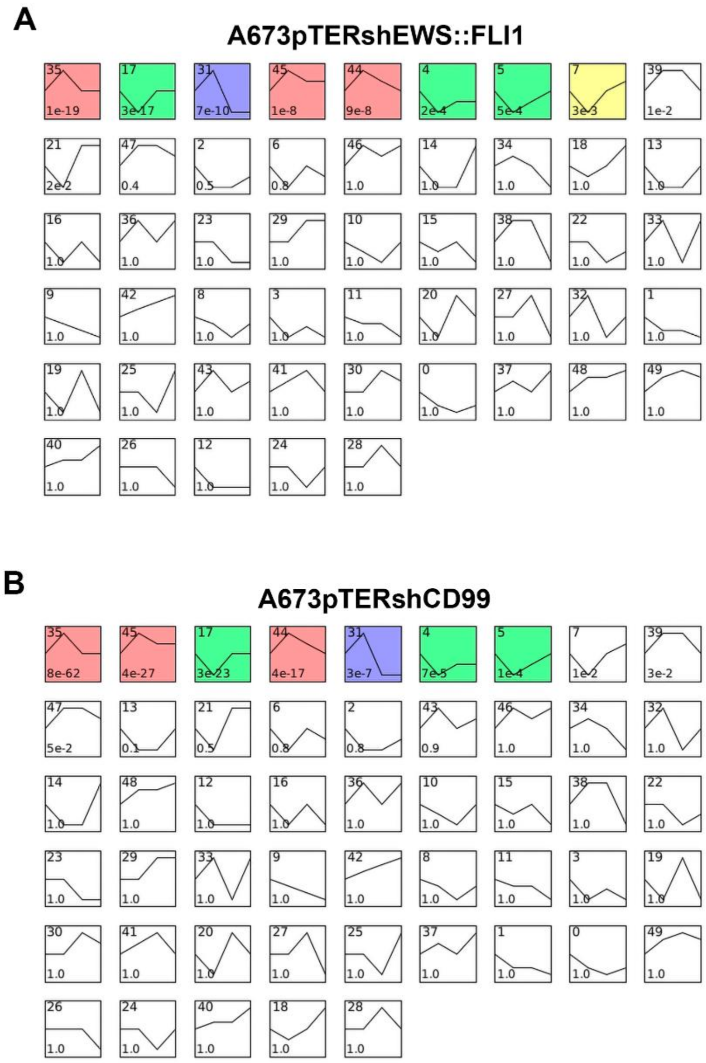

Supplementary Figure S3: Short Time-series Expression Miner (STEM) algorithm profiles overview of (**A**) A673pTERshEWS::FLI1 and (**B**) A673pTERshCD99. Clusters and profiles were ordered based on p-value significance of number of genes assigned versus expected. Profiles with the same color show similar patterns (based on correlation coefficients) and are grouped together to form a cluster of significant profiles.

Supplementary Figure S4

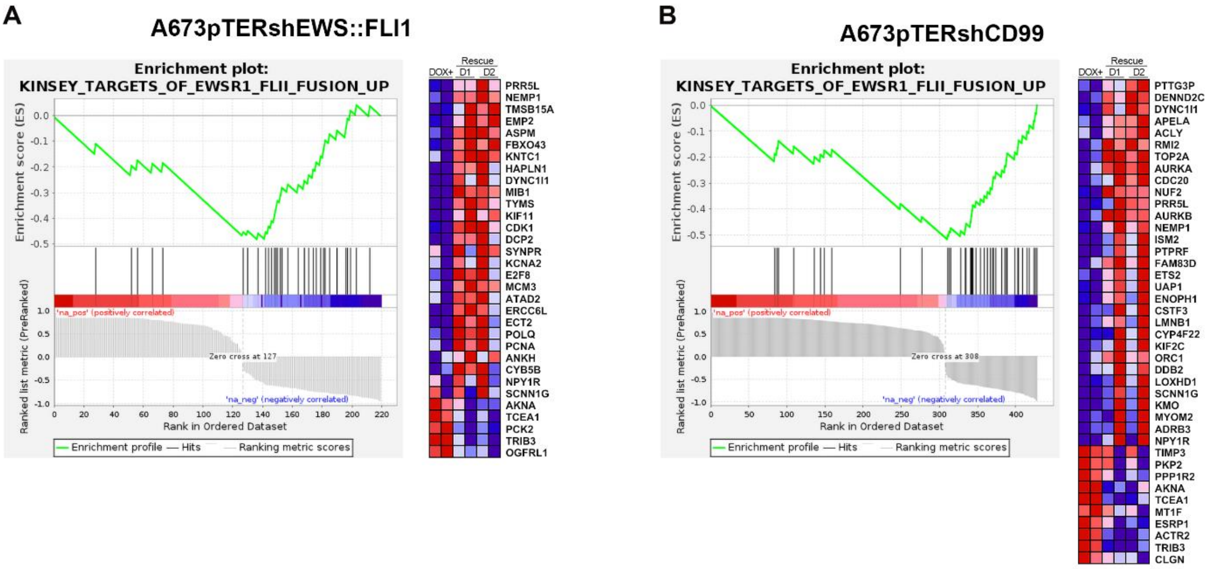

Supplementary Figure S4: GSEA revealed a significant enrichment of downregulated genes in EWS::FLI1 target genes (Kinsey and colleagues database) when EWS::FLI1 (**A**) or CD99 (**B**) expression was silenced. The enrichment score curve was obtained using GSEA software employing a continuous phenotype label and Pearson's correlation metric for ranking genes. In the enrichment plot, the x-axis shows the rank of genes from the most upregulated to the most downregulated between D0, D1 and D2. The vertical black line indicates the position of the enriched genes (Hit) comprising the gene set. The heatmaps show the genes that contribute most to the core enriched pathway; red and blue colours indicate genes up and downregulated, respectively, in D0, D1 and D2.

Supplementary Figure S5

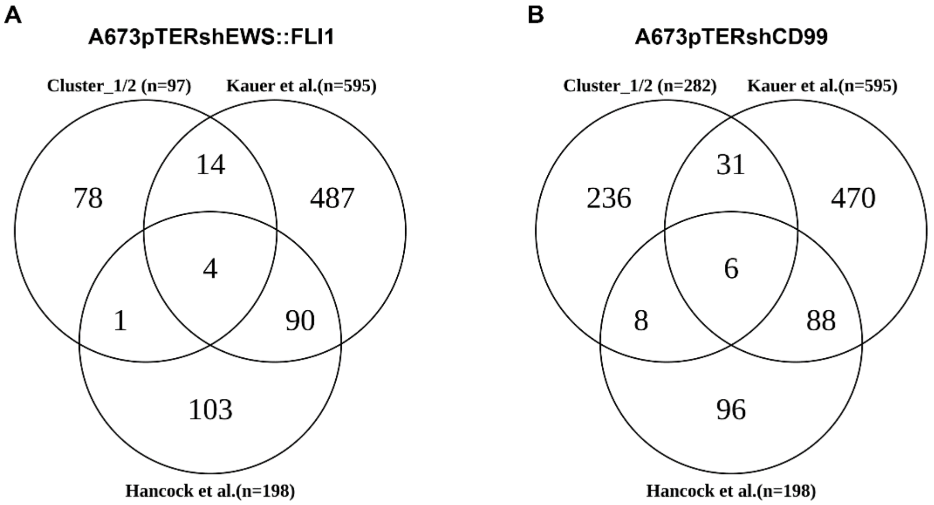

p (Fisher's exact test)	A673pTERshEWS::FLI1		A673pTERshCD99	
	Cluster_1/2 (up-regulated genes)	Odds ratios	Cluster_1/2 (up-regulated genes)	Odds ratios
Kauer et al. (up-regulated genes)	3.6e-09	6.7	1.4e-12	4.6
Hancock et al. (down-regulated genes)	4.8e-03	4.9	3.8e-06	4.9

C

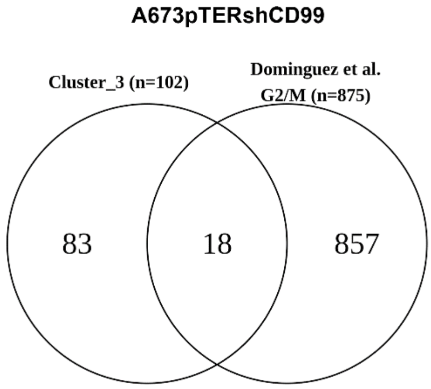

p (Fisher's exact test)	A673pTERshCD99	
	Cluster_3 (down-regulated genes)	Odds ratios
Dominguez et al.	2.2e-06	4.2

Supplementary Figure S5: Overlap of A673pTERshEWS::FLI1 and A673pTERshCD99 cluster 1/2 with two described EwS gene signatures. (**A**) Venn diagram of A673pTERshEWS::FLI1 cluster 1/2 genes with EwS up-regulated genes identified by Kauer et al. and downregulated genes identified by Hancock et al.

(**B**) Venn diagram of A673pTERshCD99 cluster 1/2 genes with EwS up-regulated genes identified by Kauer et al. and downregulated genes identified by Hancock et al.(**C**) Overlap of A673pTERshCD99 Cluster 3 with the transcriptomic signatures associated to the specific phases of the cell cycle. Venn diagram of A673pTERshCD99 cluster 3 genes with the transcriptome map of cell cycle reported by Dominguez et al. We

found a strong matching between genes enriched in DREAM complex targets and G2/M cell cycle phases. The table reports p values (Fisher test) and odds ratios.

Supplementary Figure S6

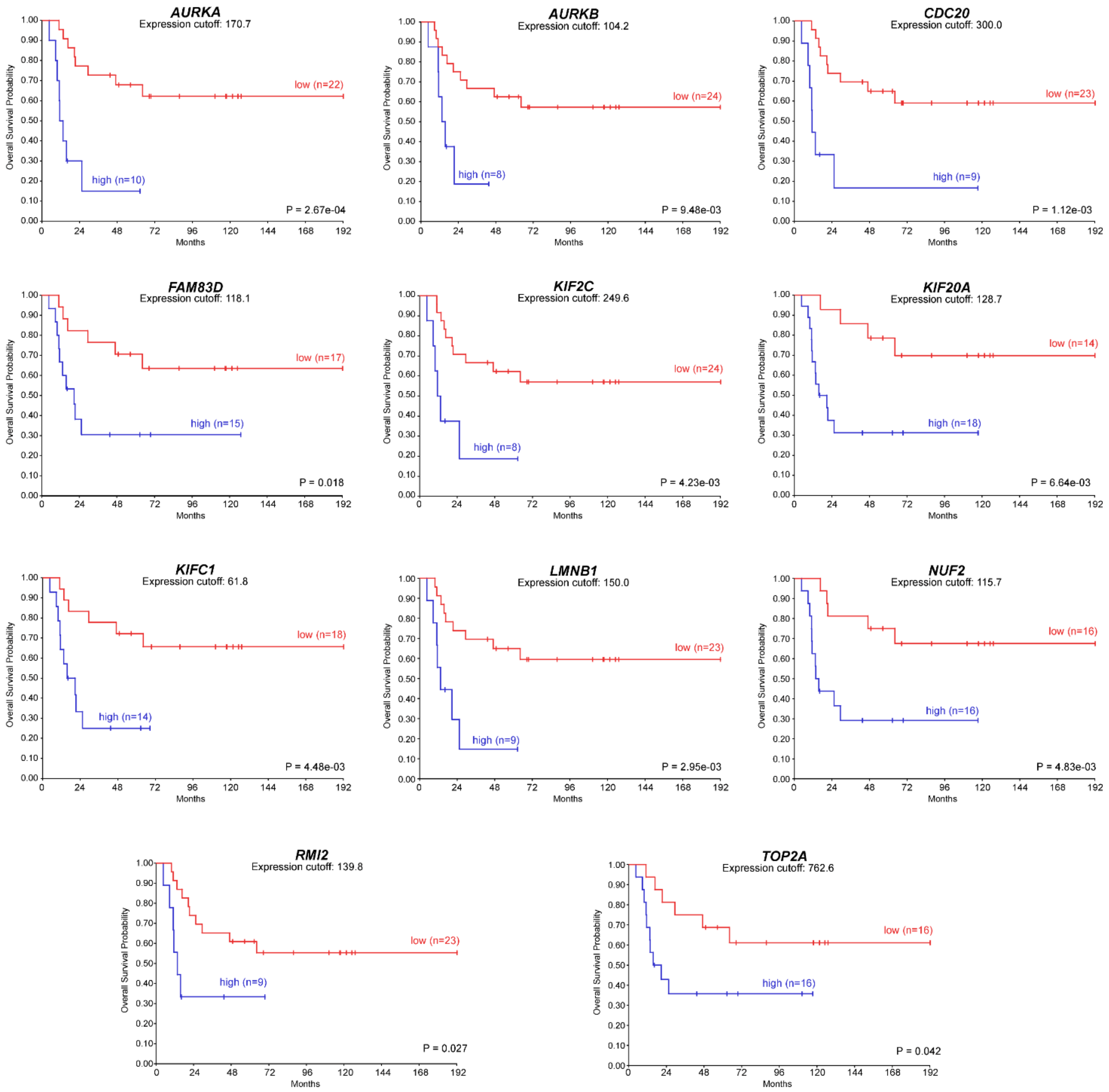

Supplementary Figure S6: Kaplan-Meier survival curves using R2 analysis software showing the overall survival (OS) probability for high (blue) versus low (red) mRNA expression of 11 DREAM complex target genes in 32 primary EwS tumors from the GSE17618 ("Mixed Ewing Sarcoma – Savola – 117 – MAS5.0 – u133p2") dataset. High gene expression levels showed a clear trend towards worse overall survival.

Supplementary Figure S7

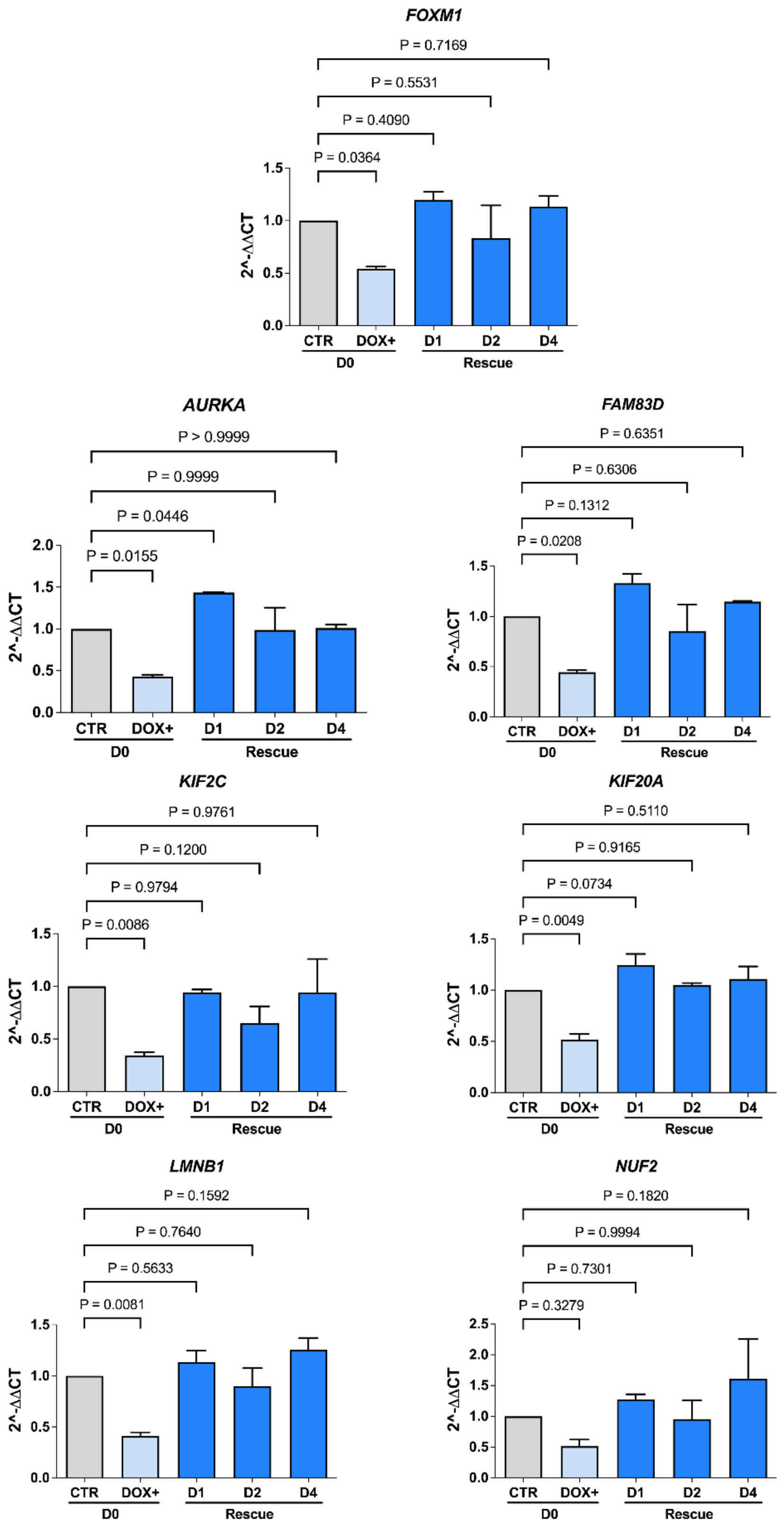

Supplementary Figure S7: Expression level of *FOXM1, AURKA, FAM83D, KIF20A, KIF2C, LMNB1* and *NUF2* by RT-qPCR in A673pTERshCD99 cells at the indicated time point. Data are expressed as mean ± SD (One-way ANOVA "Dunnett's multiple comparisons test").

Supplementary Figure S8

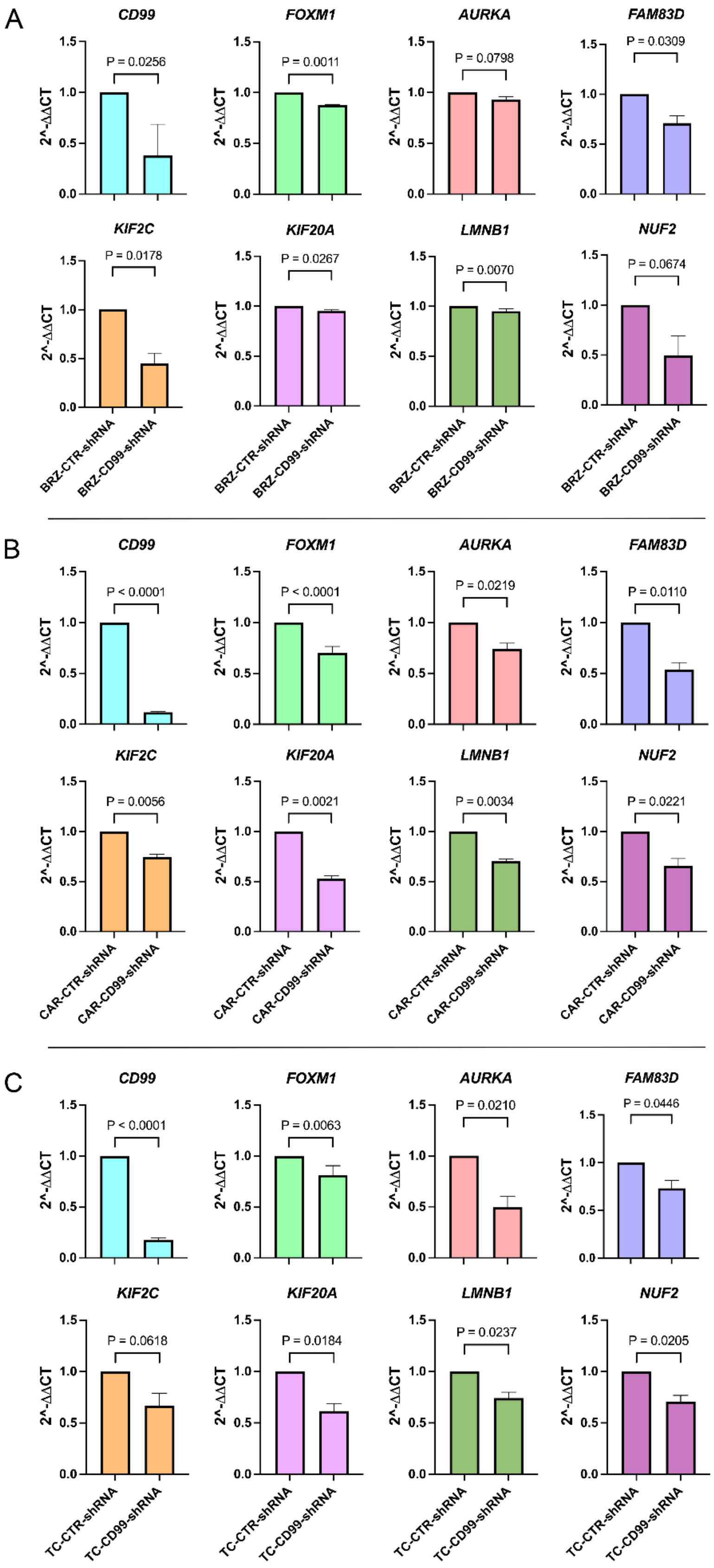

Supplementary Figure S8: Expression level of *CD99, FOXM1, AURKA, FAM83D, KIF20A, KIF2C, LMNB1* and *NUF2* by RT-qPCR in BRZ-CTR-shRNA and BRZ-CD99-shRNA cells (**A**); in CAR-CTR-shRNA and CAR-CD99-shRNA cells (**B**) and in TC-CTR-shRNA and TC-CD99-shRNA cells (**C**). Data are expressed as mean ± SD (Unpaired t test).

Supplementary Figure S9

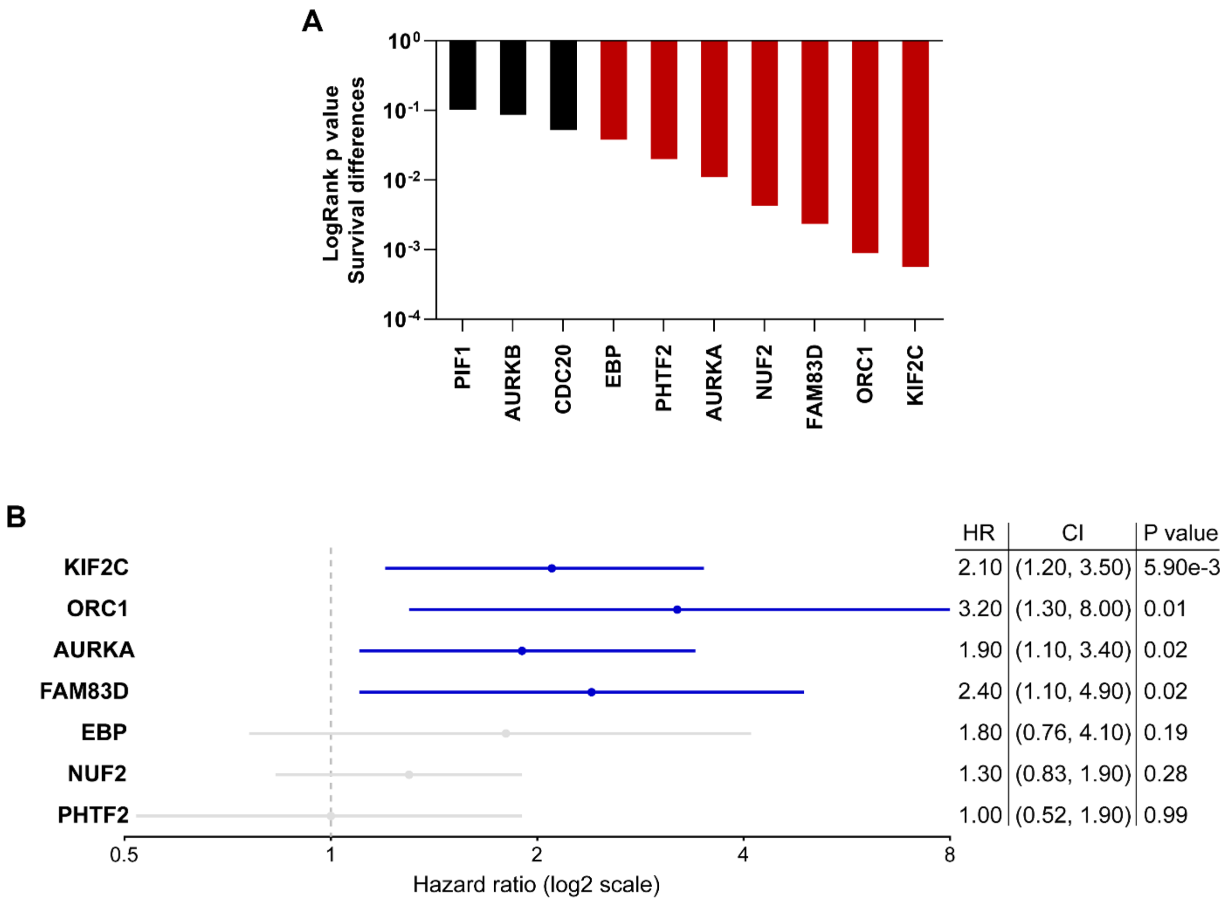

Supplementary Figure S9: Prognostic value of DREAM target genes. (**A**) Log-rank P values determined for overall survival (OS) differences based on high vs low expression of 10 CD99-modulated genes of the core DREAM gene set. "Tumor Ewing sarcoma (Core Transcript) - Dirksen - 85 - rma sketch - huex10t" was considered (GSE63157). (**B**) Multivariate Cox regression analysis identified 4 independent prognostic factors for EwS overall survival. (HR, hazard ratio; CI, confidence interval).
